# Maternal Genetic Variation Accounts in Part for the Associations of Maternal Size during Pregnancy with Offspring Cardiometabolic Risk in Adulthood

**DOI:** 10.1371/journal.pone.0091835

**Published:** 2014-03-26

**Authors:** Pandora L. Wander, Hagit Hochner, Colleen M. Sitlani, Daniel A. Enquobahrie, Thomas Lumley, Gabriela M. Lawrence, Ayala Burger, Bella Savitsky, Orly Manor, Vardiella Meiner, Stephanie Hesselson, Pui Y. Kwok, David S. Siscovick, Yechiel Friedlander

**Affiliations:** 1 Department of Epidemiology, University of Washington, Seattle, Washington, United States of America; 2 Department of Medicine, University of Washington, Seattle, Washington, United States of America; 3 Braun School of Public Health, Hebrew University-Hadassah Medical Center, Jerusalem, Israel; 4 Cardiovascular Health Research Unit, University of Washington, Seattle, Washington, United States of America; 5 Department of Statistics, University of Auckland, Auckland, New Zealand; 6 Department of Human Genetics, Hebrew University-Hadassah Medical Center, Jerusalem, Israel; 7 Institute of Human Genetics, University of California San Francisco, San Francisco, California, United States of America; 8 Cardiovascular Research Institute, University of California San Francisco, San Francisco, California, United States of America; 9 Department of Dermatology, University of California San Francisco, San Francisco, California, United States of America; Johns Hopkins Bloomberg School of Public Health, United States of America

## Abstract

**Background:**

Maternal pre-pregnancy body-mass index (ppBMI) and gestational weight gain (GWG) are associated with cardiometabolic risk (CMR) traits in the offspring. The extent to which maternal genetic variation accounts for these associations is unknown.

**Methods/Results:**

In 1249 mother-offspring pairs recruited from the Jerusalem Perinatal Study, we used archival data to characterize ppBMI and GWG and follow-up data from offspring to assess CMR, including body mass index (BMI), waist circumference, glucose, insulin, blood pressure, and lipid levels, at an average age of 32. Maternal genetic risk scores (GRS) were created using a subset of SNPs most predictive of ppBMI, GWG, and each CMR trait, selected among 1384 single-nucleotide polymorphisms (SNPs) characterizing variation in 170 candidate genes potentially related to fetal development and/or metabolic risk. We fit linear regression models to examine the associations of ppBMI and GWG with CMR traits with and without adjustment for GRS. Compared to unadjusted models, the coefficient for the association of a one-standard-deviation (SD) difference in GWG and offspring BMI decreased by 41% (95%CI −81%, −11%) from 0.847 to 0.503 and the coefficient for a 1SD difference in GWG and WC decreased by 63% (95%CI −318%, −11%) from 1.196 to 0.443. For other traits, there were no statistically significant changes in the coefficients for GWG with adjustment for GRS. None of the associations of ppBMI with CMR traits were significantly altered by adjustment for GRS.

**Conclusions:**

Maternal genetic variation may account in part for associations of GWG with offspring BMI and WC in young adults.

## Introduction

Increasingly, the enduring consequences of maternal perinatal obesity (pre-pregnancy obesity and excess gestational weight gain) are being recognized. A recent analysis by our group demonstrated associations of maternal pre-pregnancy body-mass index (BMI) and gestational weight gain (GWG) with adulthood obesity-related risk factors for metabolic and cardiovascular diseases in the offspring [1]. These findings are especially concerning given current epidemic rates of pre-pregnancy obesity and excess GWG [2], [3]. More than 20% of women are obese at the start of pregnancy [4], and more than 40% of gravidae gain in excess of Institute of Medicine recommendations [5].

Factors that account for the associations of maternal perinatal obesity with offspring cardiometabolic (CMR) traits are poorly understood. Maternal variation in genes involved in pathways related to metabolic risk and intrauterine growth may account for these associations through (1) direct transmission of genetic susceptibility to the offspring or (2) their influence on the intrauterine environment, critical in early life programming, growth, and development. Whether maternal genetic variation accounts, at least in part, for associations of maternal perinatal obesity with offspring CMR traits has not been fully examined. Previous analyses have provided limited evidence for an effect of maternal genetic variation on the association of measures of maternal perinatal obesity with offspring obesity at birth [6], in childhood [7] and in young adulthood [8] using study designs such as linear mixed models (that use clustering or other statistical techniques to account for shared maternal characteristics, including maternal genetic variation). However, although these studies tended to suggest that maternal characteristics were important in accounting for part of the association, the study designs used were unable to distinguish the effects of shared genetic from shared environmental factors. To our knowledge, there have been no published studies investigating these associations that controlled for genetic variation with direct measures of maternal or offspring genotype. Further, limited evidence suggests that genes that are reliably and strongly associated with obesity may not be important in influencing gestational weight gain [9]; therefore, maternal genetic variation may act differently in associations of offspring CMR with maternal pre-pregnancy BMI or GWG.

We sought to determine whether maternal genetic variation in a set of candidate genes thought to be associated with intrauterine growth and metabolic risk accounts, at least in part, for the associations of maternal size before and during pregnancy with CMR traits in their young adult offspring.

## Methods

### Study Design and Population

The design and implementation of the Jerusalem Perinatal Study (JPS) have been described in detail previously.[10]–[13] Briefly, the JPS is a population-based cohort comprising all 17,003 births to residents of Jerusalem between 1974 and 1976. Data include demographic and socioeconomic information, medical conditions of the mother during current and previous pregnancies, and offspring birth weight, abstracted from birth certificates and maternity ward logbooks. Information on maternal medical conditions, including gestational age, smoking status, height, and pre-pregnancy and end-of-pregnancy weights, was collected by interviews of mothers on the first or second day postpartum.

The JPS Family Follow-Up study includes a sample of 1,249 mother-offspring pairs with available clinical and genotypic data from the original 1974–1976 birth cohort, who were interviewed and examined between 2007 and 2009. The sampling frame included only singleton deliveries after 36 weeks gestation without congenital malformations. We obtained a stratified sample of eligible pairs, where the strata were defined by ppBMI and birth weight. Pairs with low (≤2500 grams) and high (≥4000 grams) birth weight offspring as well as overweight or obese mothers (BMI≥27) were over-sampled. Approximately 80% of those sampled participated in a detailed interview and clinical examination.

### Ethics Statement

The study was approved by the committee on research involving human subjects of the Hebrew University-Hadassah Medical School, by the Israeli National Genetic IRB committee, and by the institutional review board of the University of Washington, and all subjects provided written informed consent.

### Data Collection

Data on physical activity, ethnic origin, education, and smoking status were collected. Standing height in cm was measured without shoes. Body weight in kg was measured in indoor light clothes, and body mass index (BMI) was defined as kilograms divided by meters squared (kg/m^2^). Waist circumference (WC) was an average of two measurements taken at the midpoint between the lower ribs and iliac crest, and an average of resting blood pressure in the right arm was obtained in mmHg from three consecutive measurements after five minutes of rest (Omron M7 automated sphygmomanometer). Blood samples were taken after an eight-hour fast, and analyses of glucose, total cholesterol, LDL-C, HDL-C, and triglycerides are reported in mg/dL. Insulin levels were determined by radioimmunoassay using a double-antibody method (Millipore) in mU/L and reflect an average of two values.

### Genotyping

Genomic DNA from mothers and offspring was extracted and amplified at Hebrew University using conventional methods. Genotyping was performed at University of California, San Francisco using an Illumina, Inc., BeadArray. For this analysis, data on only maternal genotype were used.

We examined 1,384 single-nucleotide polymorphisms (SNPs) from 170 candidate genes in molecular pathways related to fetal development and metabolic risk in adults (Table S1). Genes selected were in pathways involved in fetal development and cardiometabolic risk, such as insulin sensitivity and signaling, neural appetite regulation, adipocyte homeostasis, and regulation of the hypothalamic-pituitary-adrenal (HPA) axis. We selected polymorphic sites representing common variation within candidate genes using available SNP data sets. We identified common tagSNPs (MAF >0.1) that capture the common variation in these candidate genes. We imputed dosages for missing SNPs using MACH 1.0 [14], [15] and discarded SNPs with more than 5% of values missing in our original sample. Tests of Hardy-Weinberg equilibrium (HWE) were performed. All but 10 SNPs were in HWE using a Chi-squared test with an alpha level of 0.05/1380 SNPs.

### Statistical Methods

For our initial analyses, we fit linear regression models to examine the associations of pre-pregnancy BMI and GWG with each CMR trait. All traits that were not normally distributed were log-transformed. All analyses used inverse probability weighting to account for our sampling scheme, and all models were adjusted for offspring sex and ethnic background. Ethnic background of offspring was classified based on country of origin of all four grandparents, using nine strata (Israel, Morocco, Other North Africa, Iran, Iraq, Kurdistan, Yemen, Other Asia and the Balkans, and Ashkenazi). [16] For each subject, we constructed nine variables (one for each stratum). The value for each variable ranged from 0 to 1, reflecting the proportion of the four grandparents that came from that ethnic group. If no grandparents came from that group, the value of the variable was set to 0. If all four came from that group, the value was set to 1. In analyses, we used Ashkenazi ethnic background as a reference group.

Additionally, we included covariates thought to be associated with the exposures and outcomes. Maternal covariates included: parity (continuous); mother’s age at childbirth (continuous); maternal smoking during the pregnancy (ever/never smoked); socioeconomic status based on father’s occupation at time of birth and categorized as low, medium, and high; mother’s years of education at time of birth (continuous); a dichotomous variable for mother’s health during pregnancy, reflecting the presence of diabetes, hypertension, or pre-eclampsia; and gestational age in weeks (continuous). Offspring covariates were birthweight (continuous); smoking status (ever/never smoked), a dichotomous variable for physical activity (based on the question: during leisure time are you engaged in moderate or vigorous physical activity that lasts at least 20 minutes, 3 or more times a week?), and years of education (continuous). Pre-pregnancy BMI models were also adjusted for GWG, and GWG models for pre-pregnancy BMI. Because of the potential that covariates that occurred after conception might mediate the associations of interest, models unadjusted for birthweight, offspring smoking status, offspring physical activity, and offspring years of education were also fit. The first part of this analysis replicates an analysis recently published in Circulation on a similar dataset [17]. Because of availability of genetic data, sample size differed slightly from this earlier group.

We created genetic risk scores, using the Lasso shrinkage and selection method [18], [19] to select the combination of maternal SNPs that was most predictive for each maternal characteristic and the combination of maternal SNPs that was most predictive for each adult offspring CMR trait. Using the SNPs chosen by the Lasso procedure, we calculated participant-level predicted values (genetic scores) for each characteristic and CMR trait. We then re-evaluated the associations of mppBMI and GWG with each offspring CMR trait in linear models that included two genetic scores, one for the maternal characteristic and one for the CMR trait, in addition to other covariates: parity, maternal age at childbirth, maternal smoking, father’s SES, mother’s education, maternal health status during pregnancy, gestational age, birthweight, offspring smoking, offspring physical activity, and offspring education. Both genetic scores were included in the models to more fully adjust for genetic information. [20] We calculated percent change in the coefficients for the associations of the maternal characteristic and the offspring CMR trait, comparing the models that did and did not adjust for maternal genetic scores, and generated bootstrap confidence intervals for the estimates of percent change.

To examine effects of variation in genes participating in these *a priori*-specified individual molecular pathways, we performed follow-up exploratory analyses. In models where a significant change was seen between coefficients unadjusted and adjusted for genetic risk score, we repeated the analyses described above, using only SNPs associated with specific molecular pathways, insulin sensitivity and signaling, neural appetite regulation, adipocyte homeostasis, and endothelial function. We repeated the Lasso procedure on these subsets to again choose a combination of SNPs that was predictive, and repeated our models with scores generated from these shorter lists of SNPs. A two-sided p-value of <0.05 was considered statistically significant. Analyses were performed with Stata (version 10.1; StataCorp, College Station, TX) and R (version 2.14; R Development Core Team, Vienna, Austria).

## Results

Characteristics of the cohort are summarized in **Table 1**. Mean pre-pregnancy BMI was 24.0 kg/m^2^ (range 15.8 to 39.7). Forty-five percent of mothers had a BMI greater than or equal to 25 kg/m^2^, while 6% had a BMI greater than or equal to 30 kg/m^2^. Mothers gained on average 11.1 kg during pregnancy (range −7 to 92). Mean maternal age at childbirth was 28 years (range 17 to 46), and mean birthweight was 3400 g (range 1480 to 6000). Overall, maternal medical conditions during the pregnancy (including gestational hypertension, pre-eclampsia, and gestational diabetes) affected 8% of pregnancies.

**Table 1:** Characteristics at birth and age 32, stratified by offspring sex.

	Women (N = 614)		Men (N = 635)		Total (N = 1249)	
**Characteristics obtained at birth**						
Maternal pre-pregnancy BMI, kg/m^2^	24.3	(3.9)	23.7	(3.9)	24.0	(3.8)
Gestational weight gain, kg	10.8	(4.6)	11.4	(4.6)	11.1	(4.6)
Maternal smoking in pregnancy, %	16.8		18.3		17.5	
Maternal ethnic origin, %						
*Israel*	12.3		13.5		13.0	
*Asia*	28.5		24.7		26.6	
*Africa*	23.0		23.5		23.2	
*West*	36.2		38.3		37.2	
Maternal years of education	11.8	(3.4)	12.0	(3.4)	11.9	(3.4)
Parity	2.9	(2.0)	2.8	(1.8)	2.9	(1.9)
Mother’s age	28.4	(5.6)	28.0	(5.2)	28.2	(5.4)
Socioeconomic status, %						
*Low*	21.1		23.4		22.3	
*Medium*	41.7		32.0		36.7	
*High*	37.1		44.6		40.9	
Birth weight, %						
*2500 g and below*	13.4		8.8		11.1	
*2501–3999 g*	70.3		58.4		64.1	
*4000 g and above*	16.6		32.8		24.8	
Gestational age at delivery, weeks	40.0	(1.5)	40.0	(1.5)	40.0	(1.5)
Mothers with any medical condition*, %	8.6		7.2		7.9	
**Characteristics obtained at age 32**						
Education, years	14.9	(2.6)	15.4	(3.6)	15.2	(3.2)
Smokers, %	18.5		35.3		27.0	
Physically active**, %	49.4		54.9		52.2	
Systolic BP, mmHg	98.8	(9.5)	113.9	(10.5)	106.4	(12.6)
Diastolic BP, mmHg	68.6	(8.0)	74.8	(7.9)	71.7	(8.6)
Waist circumference, cm	81.2	(13.3)	91.4	(12.3)	86.4	(13.8)
BMI, kg/m^2^	25.9	(5.4)	26.9	(4.8)	26.4	(5.2)
HDL-C, mg/dL	57.1	(15.0)	43.3	(10.9)	50.0	(14.8)
LDL-C, mg/dL	107.9	(28.3)	117.3	(28.2)	112.7	(28.6)
Triglycerides, mg/dL	91.9	(49.5)	121.2	(82.0)	106.9	(69.7)

Values are expressed as mean (SD) or percent.

*Diabetes, hypertension, heart disease, or pre-eclampsia.

**Includes self-report of moderate or vigorous physical activity lasting at least 20 minutes, 3 or more time a week.

Offspring mean BMI was 26.4 kg/m^2^ (range 16.9 to 52.7) and mean waist circumference was 86.4 cm (range 58 to 150) at age 32. Mean blood pressure was 107/72 mmHg. Average fasting glucose was 80 mg/dL with an average insulin level of 13 mIU/mL. Average LDL-C was 113, HDL-C was 50, and triglycerides were 107 mg/dL respectively. All cardiometabolic risk factors other than insulin and triglycerides were normally distributed.

**Table 2** presents the results of linear regression models examining the associations of maternal ppBMI and GWG with offspring cardiometabolic risk, both with and without genetic risk score terms. Coefficients show the average change in the mean level in the outcome associated with a one-standard deviation increase in maternal BMI or GWG. The coefficients of insulin and triglyceride levels (the log-transformed outcomes) have been back transformed and show the ratio of geometric mean of the outcome associated with a one-standard deviation increase in maternal pre-pregnancy BMI or GWG.

**Table 2:** Association between maternal pre-pregnancy BMI and gestational weight gain and offspring cardiometabolic phenotypes at age 32, with and without maternal genetic score contribution.

*Maternal pre-pregnancy BMI*						
Phenotype	n	Models withoutgenetic scores		Models withgenetic scores		% change in β[95% CI]
		Estimatedcoefficient	p-value	Estimatedcoefficient	p-value	
Offspring BMI(kg/m^2^)	1092	1.810	<0.0001	1.744	<0.0001	−4% [−24%, 21%]
Waistcircumference (cm)	1093	3.244	<0.0001	2.988	<0.0001	−8% [−36%, 25%]
Glucose (mg/dL)	940	0.726	0.155	0.403	0.337	−45% [−279%, 348%]
Log-transformedinsulin*	930	1.051	0.079	1.048	0.109	−5% [−124%, 482%]
Systolic bloodpressure (mmHg)	1079	1.685	0.004	1.570	0.006	−7% [−56%, 103%]
Diastolic bloodpressure (mmHg)	1079	1.195	0.011	1.195	0.015	0% [−77%, 146%]
HDL-C (mg/dL)	984	−1.661	0.022	−2.265	0.004	−37% [−262%, 27%]
LDL-C (mg/dL)	974	2.473	0.123	1.514	0.261	−39% [−182%, 316%]
Log-transformedtriglycerides (mg/dL)*	984	1.057	0.040	1.063	0.033	10% [−73%, 273%]
***Gestational weight gain***						
**Phenotype**	**n**	**Models without** **genetic scores**		**Models with** **genetic scores**		**% change in β** **[95% CI]**
		**Estimated** **coefficient**	**p-** **value**	**Estimated** **coefficient**	**p-** **value**	
**Offspring BMI** **(kg/m^2^)**	**1092**	0.847	**<0.0001**	0.503	**0.010**	**−41% [−81%, −11%]**
**Waist** **circumference (cm)**	**1093**	1.196	**0.039**	0.443	**0.370**	**−63% [−318%, −11%]**
Glucose (mg/dL)	940	0.354	0.542	0.509	0.219	44% [−99%, 1304%]
Log-transformedinsulin*	930	1.020	0.512	0.999	0.971	−105% [−1627%, 103%]
Systolic bloodpressure (mmHg)	1079	0.955	0.062	0.771	0.099	−19% [−126%, 269%]
Diastolic bloodpressure (mmHg)	1079	0.739	0.105	0.338	0.438	−54% [−514%, 13%]
HDL-C (mg/dL)	984	−1.420	0.071	−1.538	0.062	−8% [−288%, 62%]
LDL-C (mg/dL)	974	1.103	0.509	−0.647	0.634	−159% [−2168%, 32%]
Log-transformedtriglycerides (mg/dL)*	984	1.039	0.144	1.043	0.037	38% [−22%, 1007%]

All coefficients are for a 1-standard deviation change in level of the exposure variable (PBMI 1SD = 3.71, GWG 1SD = 4.61).

*Values were log-transformed to better approximate a normal distribution. To simplify interpretation, back-transformed results are presented and show the ratio of geometric mean of the outcome associated with a one-SD increase in BMI or GWG.

All models were adjusted for sex, ethnicity, offspring birthweight, maternal disease during pregnancy, parity, maternal smoking during pregnancy, family SES during pregnancy, gestational weeks at birth, maternal age at pregnancy, maternal education, offspring physical activity, offspring smoking status, and offspring education.

Pre-pregnancy BMI models were also adjusted for gestational weight gain, and GWG models for BMI.

All models used inverse probability weighting to account for stratified sampling.

In the models with scores, genetic scores predicting exposure and outcome are included. Scores were generated using the lasso algorithm, incorporating SNPs with no more than 5% missingness.

Confidence intervals for the % change in β were obtained via bootstrapping and based on quantiles of bootstrap replicates.

As reported previously [1], in the models without genetic scores, there were significant associations between pre-pregnancy BMI and offspring BMI (β = 1.810, p<0.0001), waist circumference (WC) (β = 3.244, p<0.0001), systolic (β = 1.685, p = 0.004) and diastolic blood pressure (β = 1.195, p = 0.011), HDL-C (β = −1.661, p = 0.022), and log-transformed triglycerides (e^β^ = 1.057, p = 0.040). There were also significant associations between GWG and offspring BMI (β = 0.847, p<0.0001)), as well as GWG and offspring WC (β = 1.196, p = 0.039) (**Table 2**).

Compared to models not adjusted for maternal genetic risk scores, the coefficient for the association of a one-standard deviation difference in GWG and offspring BMI decreased by 41% (95% CI −81%, −11%) from 0.847 to 0.503 and the coefficient for a one-standard deviation in GWG and WC decreased by 63% (95% CI −318%, −11%) from 1.196 to 0.443, with adjustment for the maternal genetic risk scores. For the association of GWG and offspring BMI, the amount of variability explained by the model without maternal genetic risk scores was 0.16. The increase in the amount of variability explained by the model was 0.22 when maternal genetic risk score terms were included. For the association of GWG and offspring WC, the amount of variability explained by the model without GRS was 0.29. The increase in the amount of variability was 0.16 when GRS terms were included.

For the other offspring CMR traits, there were no statistically significant changes in the coefficients for GWG with adjustment for the maternal genetic risk scores, and none of the associations of ppBMI and offspring CMR traits were significantly altered by adjustment of the maternal genetic risk scores. Additionally, we repeated the analyses excluding covariates that occurred after the exposure of interest (including birthweight, offspring smoking status, offspring education level, and offspring physical activity level). For example, in the association of maternal gestational weight gain with offspring BMI, with these offspring covariates *excluded,* we model a 0.857 kg/m2 for each one–standard-deviation increase in gestational weight gain, and the coefficient is 45% lower with genetic terms (95% CI −84%, −16%). Similarly, there are no significant differences in any of the other associations comparing models with these offspring covariates included or excluded. Lastly, in analyses using genetic risk scores generated from our *a priori* lists of SNPs associated with four pathways–insulin sensitivity and signaling, neural appetite regulation, adipocyte homeostasis, and endothelial function–none of the associations of maternal size and offspring CMR traits were significantly altered with inclusion of maternal genetic risk score terms.

## Discussion

In this study, associations of GWG with adult offspring measures of central adiposity (waist circumference) and overall adiposity (BMI) were attenuated in models that accounted for variation in maternal genes associated with fetal development and metabolic risk. In contrast, associations of maternal ppBMI with offspring BMI and WC were not similarly attenuated when we accounted for variation in a similar set of maternal genes. The observed attenuation of the GWG-offspring body size association by maternal genetic variation was independent of ppBMI.

Previous large-scale epidemiologic studies without direct measurement of genetic information have used statistical techniques such as generalized-estimating equations or linear mixed models to explore the role of maternal genetic variation in maternal size-offspring CMR associations, specifically offspring size, at various time points[6]–[8]. In general, previous study results suggest an important role for shared maternal characteristics (which include maternal genetic variation) in maternal-offspring size associations. However, while these analyses allow inference about the importance of shared maternal characteristics, the study designs cannot distinguish the effects of shared genetic from shared environmental factors. Our analysis is the first, to our knowledge, to use direct measurements of maternal genotype. In addition, the datasets available for previous studies have been limited by lack of adjustment for important confounders (such as pre-pregnancy BMI in some cases), while nearly complete descriptive data were available in this cohort.

Several reasons may explain the observation that variation in a set of maternal genes associated with development and metabolic risk appear to account for–at least in part–the associations of GWG with measures of offspring body size but not similar associations of ppBMI with offspring size. Firstly, the relationships may be different because the exposures themselves measure very different constructs. Pre-pregnancy BMI is a measure of baseline maternal obesity, while GWG is a surrogate for a complex measure–change in maternal body composition over the course of pregnancy–and incorporates contributions from both maternal diet and physical activity [21]. Dietary intake patterns are associated with changes in pathways governing regulation of appetite, neuroendocrine functioning, and metabolism in the fetus [22], [23]. Evidence regarding energy expenditure is more limited, but also suggests associations between maternal physical activity and measures of autonomic control and neuroendocrine function in offspring [24]. In humans, it has proven challenging to differentiate between effects due to baseline maternal obesity and effects due to change in maternal size, but in animal models, there is evidence that, independent of maternal pre-pregnancy body size, overnutrition (excess exposure to glucose, amino acids, and free fatty acids) *in utero* may result in permanent changes in appetite control, neuroendocrine functioning and/or energy metabolism in offspring [23], [25], [26], [27]. GWG and pre-pregnancy BMI appear to have different genetic determinants as well [9].

Conversely, the lack of attenuation of association between ppBMI and offspring body size by maternal genetic variation is surprising given the high heritability of obesity, but there are possible explanations. Because GWG can be influenced by both maternal and fetal contributions, accounting for common maternal genetic variation may reflect genetic influences from mother *or* fetus. Because the IUE milieu is influenced by contributions from both maternal and offspring genes, as well as environmental factors, the attenuation of the GWG-offspring body size relationship could point to a direct effect of maternal genotype on the IUE, to genes transmitted to the offspring that affect the IUE, or to a combination of both. This phenomenon is not present in the ppBMI-offspring size association. A recent analysis from the JPS cohort supports this possibility. In that study, which looked at associations of GWG and ppBMI with longitudinal change in offspring BMI, offspring genetic risk score accounted for part of the association of GWG with offspring change in BMI over time [10]. A similar effect of offspring genetic risk score was not seen in the association of ppBMI with offspring phenotype.

Lastly, epigenetic changes may amplify, attenuate, or mediate the effect of genotype on maternal GWG-offspring CMR associations. Siblings born before and after mothers underwent gastric bypass demonstrated differential levels of methylation of genes in glucoregulatory and inflammatory pathways [28]. Also, a recent analysis showed candidate gene methylation levels of DNA in umbilical cord blood was associated with both maternal high-carbohydrate diet during pregnancy and offspring size in childhood [29]. Of value to this analysis, there is some evidence that DNA mutations may impact patterns of DNA methylation [30], therefore, the role of genotype in GWG-CMR associations could be amplified by sequence-dependent epigenetic changes, but the association of GWG or ppBMI with sequence-dependent epigenetic changes has not been investigated.

This study has two major strengths: First, it uses detailed records of maternal and offspring characteristics obtained at birth and in young adulthood. This allowed for adjustment for pregnancy-related factors and socioeconomic characteristics. Second, unlike previous studies, which have used statistical techniques to estimate the contribution of maternal genetic variation to associations of maternal and offspring size [7], [8], we are able to use direct measures of maternal genotype. Our study has some potential limitations, however. Information on potentially important environmental factors, such as maternal dietary intake and physical activity during pregnancy, and physiologic values, such as gravid measures of glycemia, lipemia, and inflammation, was not available in this dataset. It is also possible that residual confounding by population stratification still remains. Gestational weight gain, given that it includes contributions from increases in maternal plasma volume, amniotic fluid, placenta, and the fetus itself [31], is also a limited proxy for increase in maternal adiposity during pregnancy. By conservative estimates, we cannot exclude the possibility that the multiple statistical tests performed could increase the possibility of a type I error, however, it is the change in coefficients and their bootstrapped confidence intervals, and not the multiple point estimates presented in Table 1 that are the primary outcome of this analysis. We cannot exclude the possibility that we were underpowered to detect significant associations in secondary analyses. Our method of using Lasso-selected SNPs to create genetic scores does not permit ascertainment of the role of specific polymorphisms. Although we selected representative single nucleotide polymorphisms in high linkage disequilibrium (tag SNPs) in each gene, we were limited to SNPs available on the experimental platform we used; therefore, gene coverage may vary. We expect that for smaller genes and genes without much recombination, the coverage will be good. Lastly, our list of polymorphisms was not selected from positive GWAS hits, and, although extensive, may not include all relevant loci. Given the relatively poor performance of candidate gene studies at detecting gene-disease associations, it is possible that if validated GWAS SNPs for obesity or other CMR risk traits had been used our reported results might have been even stronger.

Better understanding of these mechanisms is critical to guide recommendations for healthy gestational weight gain. This analysis suggests several future avenues for investigation. First, our results require validation. Second, the role of maternal genotype in gestational weight gain has not been well established, and limited evidence suggests that SNPs strongly associated with adult body size in genome-wide analyses may not be associated with higher levels of GWG [9], [32]. These findings suggest that different genetic variations may be related to ppBMI or GWG. The attenuation of the GWG-offspring size association was not seen when we looked individually at subsets of SNPs related to insulin sensitivity and signaling, neural appetite regulation, adipocyte homeostasis, and endothelial function, although an overall effect was seen looking at all SNPs together. This may be due to our methods of SNP selection, and future analyses using novel pathway-level techniques may be revealing. Lastly, this analysis was unable to capture any potential epigenetic effects. Studies investigating epigenetic mechanisms governing DNA expression in the setting of maternal overnutrition are also potential targets.

## Supporting Information

Table S1 Single nucleotide polymorphisms used to calculate maternal genetic risk scores.
(DOCX)
