# Supplementary material for: Maternal Genetic Variation Accounts in Part for the Associations of Maternal Size during Pregnancy with Offspring Cardiometabolic Risk in Adulthood
Source: PLoS One. 2014 Mar 26;9(3):e91835. doi: 10.1371/journal.pone.0091835 (PMC3966761; doi:10.1371/journal.pone.0091835)
Supplement: Table S1 — Single nucleotide polymorphisms used to calculate maternal genetic risk scores. (DOCX) [file pone.0091835.s001.docx]

| **Table S1. Single nucleotide polymorphisms used to calculate maternal genetic risk scores** | | | | | | |  |  |
| --- | --- | --- | --- | --- | --- | --- | --- | --- |
| **Nearest gene** | **rs#** | **Chromosome** | **Position** | **Mean allele frequency** | **Gene ID** | **Location** |  |  |
| Angiotensin-converting enzyme | rs12451328 | 17 | 58950280 | 0.48 | 1636 | intron |  |  |
|  | rs4267385 | 17 | 58937488 | 0.34 | 1636 | intron |  |  |
|  | rs4329 | 17 | 58917190 | 0.35 | 1636 | intron |  |  |
|  | rs4461142 | 17 | 58931780 | 0.41 | 1636 | intron |  |  |
|  | rs8066276 | 17 | 58942997 | 0.30 | 1636 | intron |  |  |
| Acid phosphatase 5 | rs2071484 | 19 | 11549460 | 0.47 | 54 | 5UTR |  |  |
|  | rs2305799 | 19 | 11548351 | 0.15 | 54 | coding |  |  |
| Adiponectin | rs12495941 | 3 | 188050874 | 0.43 | 9370 | intron |  |  |
|  | rs1501299 | 3 | 188053817 | 0.29 | 9370 | intron |  |  |
|  | rs182052 | 3 | 188043476 | 0.30 | 9370 | intron |  |  |
|  | rs3774261 | 3 | 188054253 | 0.49 | 9370 | intron |  |  |
|  | rs3774262 | 3 | 188054508 | 0.20 | 9370 | intron |  |  |
|  | rs3821799 | 3 | 188054180 | 0.47 | 9370 | intron |  |  |
|  | rs822391 | 3 | 188046497 | 0.16 | 9370 | intron |  |  |
|  | rs822396 | 3 | 188049571 | 0.16 | 9370 | intron |  |  |
| Adiponectin receptor 1 | rs10753929 | 1 | 201189801 | 0.07 | 51094 | flanking_5UTR |  |  |
|  | rs12733285 | 1 | 201188663 | 0.46 | 51094 | flanking_5UTR |  |  |
|  | rs1342387 | 1 | 201180979 | 0.45 | 51094 | intron |  |  |
|  | rs1539355 | 1 | 201190703 | 0.30 | 647243 | flanking_5UTR |  |  |
|  | rs16850799 | 1 | 201186474 | 0.20 | 51094 | intron |  |  |
| Adiponectin receptor 2 | rs1044471 | 12 | 1767217 | 0.47 | 79602 | 3UTR |  |  |
|  | rs10773983 | 12 | 1701507 | 0.38 | 79602 | intron |  |  |
|  | rs11061973 | 12 | 1736197 | 0.16 | 729097 | flanking_3UTR |  |  |
|  | rs11612383 | 12 | 1701616 | 0.33 | 79602 | intron |  |  |
|  | rs2058112 | 12 | 1732419 | 0.11 | 79602 | intron |  |  |
|  | rs4766415 | 12 | 1748415 | 0.46 | 79602 | flanking_5UTR |  |  |
|  | rs7132033 | 12 | 1676357 | 0.31 | 79602 | intron |  |  |
|  | rs7975600 | 12 | 1685513 | 0.16 | 79602 | intron |  |  |
| β1 adrenergic receptor | rs1801253 | 10 | 115795046 | 0.37 | 153 | coding |  |  |
| β2 adrenergic receptor | rs1042718 | 5 | 148187110 | 0.21 | 154 | coding |  |  |
|  | rs1042719 | 5 | 148187640 | 0.34 | 154 | coding |  |  |
| β3 adrenergic receptor | rs10481461 | 8 | 37937805 | 0.05 | 155 | flanking_3UTR |  |  |
|  | rs4994 | 8 | 37942955 | 0.05 | 155 | coding |  |  |
|  | rs4998 | 8 | 37940643 | 0.04 | 155 | 3UTR |  |  |
|  | rs9694197 | 8 | 37939250 | 0.05 | 155 | flanking_3UTR |  |  |
| Angiotensinogen | rs11122576 | 1 | 228913302 | 0.04 | 183 | intron |  |  |
|  | rs2004776 | 1 | 228915325 | 0.22 | 183 | intron |  |  |
|  | rs2148582 | 1 | 228916422 | 0.49 | 183 | intron |  |  |
|  | rs2478523 | 1 | 228908132 | 0.44 | 183 | intron |  |  |
|  | rs2478545 | 1 | 228910744 | 0.31 | 183 | intron |  |  |
|  | rs2493132 | 1 | 228910180 | 0.34 | 183 | intron |  |  |
|  | rs3889728 | 1 | 228915454 | 0.21 | 183 | intron |  |  |
|  | rs5050 | 1 | 228916509 | 0.19 | 183 | 5UTR |  |  |
|  | rs7079 | 1 | 228904954 | 0.37 | 183 | 3UTR |  |  |
|  | rs7539020 | 1 | 228915813 | 0.36 | 183 | intron |  |  |
| Angiotensin II receptor, type 1 | rs1492099 | 3 | 149920193 | 0.23 | 185 | intron |  |  |
|  | rs2131127 | 3 | 149906833 | 0.32 | 185 | intron |  |  |
|  | rs2638363 | 3 | 149901883 | 0.27 | 185 | intron |  |  |
|  | rs3772616 | 3 | 149920881 | 0.19 | 185 | intron |  |  |
|  | rs385338 | 3 | 149931846 | 0.26 | 185 | intron |  |  |
|  | rs389566 | 3 | 149929072 | 0.32 | 185 | intron |  |  |
|  | rs5182 | 3 | 149942085 | 0.39 | 185 | coding |  |  |
|  | rs6801836 | 3 | 149938227 | 0.29 | 185 | intron |  |  |
| Angiotensin II receptor, type 2 | rs1403543 | X | 115216220 | 0.45 | 186 | intron |  |  |
|  | rs3736556 | X | 115216363 | 0.23 | 186 | intron |  |  |
| α2-HS-glycoprotein | rs2070633 | 3 | 187818635 | 0.43 | 197 | intron |  |  |
|  | rs2593813 | 3 | 187815265 | 0.20 | 197 | intron |  |  |
|  | rs4831 | 3 | 187813663 | 0.24 | 197 | coding |  |  |
| v-akt murine thymoma viral oncogene homolog 2 | rs16974157 | 19 | 45464764 | 0.16 | 208 | intron |  |  |
|  | rs1991823 | 19 | 45472165 | 0.43 | 208 | intron |  |  |
|  | rs7250897 | 19 | 45475073 | 0.34 | 208 | intron |  |  |
| Alkaline phosphatase, placental | rs3762526 | 2 | 232951182 | 0.06 | 250 | flanking_5UTR |  |  |
| Apolipoprotein B | rs1042031 | 2 | 21079258 | 0.16 | 338 | coding |  |  |
|  | rs11126598 | 2 | 21093869 | 0.30 | 338 | intron |  |  |
|  | rs11676704 | 2 | 21097863 | 0.13 | 338 | intron |  |  |
|  | rs12714264 | 2 | 21119023 | 0.21 | 338 | intron |  |  |
|  | rs1367117 | 2 | 21117405 | 0.20 | 338 | coding |  |  |
|  | rs1469513 | 2 | 21113067 | 0.31 | 338 | intron |  |  |
|  | rs3791981 | 2 | 21098872 | 0.22 | 338 | intron |  |  |
|  | rs673548 | 2 | 21091049 | 0.25 | 338 | intron |  |  |
|  | rs679899 | 2 | 21104419 | 0.44 | 338 | coding |  |  |
|  | rs693 | 2 | 21085700 | 0.35 | 338 | coding |  |  |
| Agouti signaling protein | rs819162 | 20 | 32315943 | 0.19 | 434 | intron |  |  |
| ATG9 autophagy related 9 homolog B | rs2373929 | 7 | 150345745 | 0.35 | 285973 | intron |  |  |
|  | rs7830 | 7 | 150340504 | 0.33 | 285973 | 3UTR |  |  |
| ATPase, H+ transporting, lysosomal 38kDa, V0 subunit d1 | rs28582606 | 16 | 67475932 | N/A | 285973 | intron |  |  |
|  | rs4360931 | 16 | 66029427 | 0.12 | 9114 | 3UTR |  |  |
|  | rs749242 | 16 | 66049072 | 0.04 | 9114 | intron |  |  |
|  | rs8044652 | 16 | 66063748 | 0.50 | 9114 | intron |  |  |
|  | rs9922476 | 16 | 66036425 | 0.09 | 9114 | intron |  |  |
| Arginine vasopressin | rs2282018 | 20 | 3012949 | 0.38 | 551 | intron |  |  |
|  | rs2740185 | 20 | 3009436 | 0.18 | 551 | flanking_3UTR |  |  |
| Arginine vasopressin receptor 1A | rs11174811 | 12 | 61826743 | 0.15 | 552 | 3UTR |  |  |
| BCL2-associated X protein | rs1009316 | 19 | 54150382 | 0.12 | 581 | intron |  |  |
| B-cell CLL/lymphoma 2 | rs1026825 | 18 | 58971255 | 0.48 | 596 | intron |  |  |
|  | rs10460157 | 18 | 59123698 | 0.40 | 596 | intron |  |  |
|  | rs12967026 | 18 | 59034281 | 0.23 | 596 | intron |  |  |
|  | rs1381548 | 18 | 59108376 | 0.47 | 596 | intron |  |  |
|  | rs1531697 | 18 | 58957586 | 0.16 | 596 | intron |  |  |
|  | rs1542578 | 18 | 58965895 | 0.42 | 596 | intron |  |  |
|  | rs1564483 | 18 | 58945634 | 0.22 | 596 | 3UTR |  |  |
|  | rs17757541 | 18 | 59030666 | 0.20 | 596 | intron |  |  |
|  | rs1801018 | 18 | 59136859 | 0.40 | 596 | coding |  |  |
|  | rs1982673 | 18 | 58948995 | 0.18 | 596 | intron |  |  |
|  | rs2046136 | 18 | 58964961 | 0.34 | 596 | intron |  |  |
|  | rs2051423 | 18 | 59132236 | 0.48 | 596 | intron |  |  |
|  | rs2849380 | 18 | 59130340 | 0.28 | 596 | intron |  |  |
|  | rs2850761 | 18 | 59117377 | 0.48 | 596 | intron |  |  |
|  | rs3810027 | 18 | 59054958 | 0.32 | 596 | intron |  |  |
|  | rs4940574 | 18 | 58988824 | 0.20 | 596 | intron |  |  |
|  | rs4940576 | 18 | 58999619 | 0.30 | 596 | intron |  |  |
|  | rs4941185 | 18 | 58974534 | 0.34 | 596 | intron |  |  |
|  | rs4941189 | 18 | 59058907 | 0.32 | 596 | intron |  |  |
|  | rs4987736 | 18 | 59091298 | 0.43 | 596 | intron |  |  |
|  | rs4987768 | 18 | 59055497 | 0.23 | 596 | intron |  |  |
|  | rs4987808 | 18 | 58978566 | 0.35 | 596 | intron |  |  |
|  | rs4987825 | 18 | 58963493 | 0.08 | 596 | intron |  |  |
|  | rs4987828 | 18 | 58957852 | 0.28 | 596 | intron |  |  |
|  | rs4987829 | 18 | 58957394 | 0.26 | 596 | intron |  |  |
|  | rs4987853 | 18 | 58944635 | 0.25 | 596 | 3UTR |  |  |
|  | rs720321 | 18 | 59033630 | 0.19 | 596 | intron |  |  |
|  | rs7230970 | 18 | 58972056 | 0.47 | 596 | intron |  |  |
|  | rs7240326 | 18 | 59068331 | 0.48 | 596 | intron |  |  |
|  | rs7242402 | 18 | 59092938 | 0.08 | 596 | intron |  |  |
|  | rs7242542 | 18 | 59052906 | 0.11 | 596 | intron |  |  |
|  | rs7243091 | 18 | 59031542 | 0.22 | 596 | intron |  |  |
|  | rs8083946 | 18 | 59056901 | 0.39 | 596 | intron |  |  |
|  | rs8084922 | 18 | 59081501 | 0.49 | 596 | intron |  |  |
|  | rs8085707 | 18 | 59080760 | 0.07 | 596 | intron |  |  |
|  | rs8086404 | 18 | 59068104 | 0.27 | 596 | intron |  |  |
|  | rs8094315 | 18 | 59087027 | 0.18 | 596 | intron |  |  |
|  | rs8096380 | 18 | 59071461 | 0.23 | 596 | intron |  |  |
|  | rs9972996 | 18 | 59072870 | 0.21 | 596 | intron |  |  |
|  | rs12454712 | 18 | 60845884 | 0.40 | 596 | intron |  |  |
| Bombesin-like receptor 3 | rs1203607 | X | 135401059 | 0.33 | 680 | intron |  |  |
| Chromosome 1 open reading frame 167 | rs12564559 | 1 | 11748131 | 0.10 | 284498 | intron |  |  |
|  | rs1537514 | 1 | 11770655 | 0.09 | 284498 | coding |  |  |
|  | rs4845875 | 1 | 11746720 | 0.44 | 284498 | intron |  |  |
|  | rs4845877 | 1 | 11746890 | 0.43 | 284498 | intron |  |  |
|  | rs6541001 | 1 | 11759954 | 0.05 | 284498 | intron |  |  |
|  | rs6696752 | 1 | 11758522 | 0.39 | 284498 | intron |  |  |
| Calmodulin 2 | rs11125126 | 2 | 47248883 | 0.19 | 805 | intron |  |  |
|  | rs815815 | 2 | 47252568 | 0.08 | 805 | intron |  |  |
| G protein-coupled receptor 35 | rs2953161 | 2 | 241194074 | 0.38 | 11132 | intron |  |  |
|  | rs2975759 | 2 | 241179466 | 0.09 | 11132 | intron |  |  |
|  | rs3828345 | 2 | 241185492 | 0.14 | 11132 | intron |  |  |
| CART prepropeptide | rs3763154 | 5 | 71049233 | 0.47 | 9607 | flanking_5UTR |  |  |
|  | rs4991862 | 5 | 71049896 | 0.16 | 9607 | flanking_5UTR |  |  |
| Caspase 1, apoptosis-related cysteine peptidase | rs2282659 | 11 | 104402647 | 0.17 | 834 | intron |  |  |
|  | rs556205 | 11 | 104403218 | 0.10 | 834 | intron |  |  |
|  | rs568910 | 11 | 104409780 | 0.14 | 834 | intron |  |  |
|  | rs11226593 | 11 | 104900822 | N/A | 834 | intron |  |  |
| Caspase 3, apoptosis-related cysteine peptidase | rs1049253 | 4 | 185785945 | 0.23 | 836 | 3UTR |  |  |
|  | rs2705897 | 4 | 185790092 | 0.24 | 836 | intron |  |  |
|  | rs2705901 | 4 | 185796481 | 0.16 | 836 | intron |  |  |
|  | rs2720376 | 4 | 185791294 | 0.47 | 836 | intron |  |  |
|  | rs2720378 | 4 | 185805107 | 0.36 | 836 | intron |  |  |
|  | rs4647609 | 4 | 185804956 | 0.19 | 836 | intron |  |  |
| Cholecystokinin | rs10865918 | 3 | 42278315 | 0.33 | 885 | intron |  |  |
|  | rs1868522 | 3 | 42279788 | 0.41 | 885 | intron |  |  |
|  | rs747455 | 3 | 42280968 | 0.21 | 885 | intron |  |  |
| Cholecystokinin A receptor | rs2000978 | 4 | 26097087 | 0.14 | 886 | intron |  |  |
|  | rs2725307 | 4 | 26098155 | 0.33 | 886 | intron |  |  |
|  | rs7665027 | 4 | 26096007 | 0.23 | 886 | intron |  |  |
| Cholecystokinin B receptor | rs11040832 | 11 | 6244494 | 0.27 | 887 | intron |  |  |
|  | rs12364575 | 11 | 6248235 | 0.27 | 887 | intron |  |  |
|  | rs2929183 | 11 | 6239384 | 0.22 | 887 | intron |  |  |
|  | rs2941025 | 11 | 6239044 | 0.18 | 887 | intron |  |  |
|  | rs2941029 | 11 | 6241793 | 0.48 | 887 | intron |  |  |
|  | rs2947025 | 11 | 6239652 | 0.20 | 887 | intron |  |  |
|  | rs7943852 | 11 | 6241704 | 0.30 | 887 | intron |  |  |
| CD14 molecule | rs2563298 | 5 | 139991499 | 0.31 | 929 | flanking_3UTR |  |  |
|  | rs2569190 | 5 | 139993100 | 0.47 | 929 | 5UTR |  |  |
|  | rs5744455 | 5 | 139993491 | 0.20 | 929 | flanking_5UTR |  |  |
| CD36 molecule | rs10499858 | 7 | 80073782 | 0.08 | 948 | intron |  |  |
|  | rs1334511 | 7 | 80104004 | 0.06 | 948 | intron |  |  |
|  | rs1527483 | 7 | 80139436 | 0.06 | 948 | intron |  |  |
|  | rs1537593 | 7 | 80091847 | 0.09 | 948 | intron |  |  |
|  | rs1953299 | 7 | 80103561 | 0.48 | 948 | intron |  |  |
|  | rs3173804 | 7 | 80137786 | 0.47 | 948 | intron |  |  |
|  | rs3211908 | 7 | 80131852 | 0.05 | 948 | intron |  |  |
|  | rs997906 | 7 | 80117771 | 0.40 | 948 | intron |  |  |
| Cyclin-dependent kinase inhibitor 2A | rs2811708 | 9 | 21963422 | 0.25 | 1029 | intron |  |  |
|  | rs2811709 | 9 | 21970151 | 0.09 | 1029 | intron |  |  |
|  | rs3731204 | 9 | 21977584 | 0.12 | 1029 | intron |  |  |
|  | rs3731239 | 9 | 21964218 | 0.29 | 1029 | intron |  |  |
|  | rs4074785 | 9 | 21971583 | 0.08 | 1029 | intron |  |  |
| Cyclin-dependent kinase inhibitor 2B | rs1063192 | 9 | 21993367 | 0.28 | 1030 | 3UTR |  |  |
|  | rs2069422 | 9 | 21998026 | 0.09 | 1030 | intron |  |  |
|  | rs3217986 | 9 | 21995330 | 0.07 | 1030 | 3UTR |  |  |
|  | rs3217992 | 9 | 21993223 | 0.46 | 1030 | 3UTR |  |  |
| CCAAT/enhancer binding protein α | rs12691 | 19 | 38482967 | 0.15 | 1050 | 3UTR |  |  |
| Chromogranin B | rs13036884 | 20 | 5844721 | 0.07 | 1114 | intron |  |  |
|  | rs236143 | 20 | 5844293 | 0.16 | 1114 | intron |  |  |
|  | rs2821 | 20 | 5853779 | 0.39 | 1114 | 3UTR |  |  |
|  | rs910122 | 20 | 5851323 | 0.33 | 1114 | coding |  |  |
| Corticotropin releasing hormone | rs3176921 | 8 | 67253933 | 0.08 | 1392 | flanking_5UTR |  |  |
| Corticotropin releasing hormone binding protein | rs7718461 | 5 | 76293804 | 0.48 | 1393 | intron |  |  |
|  | rs7721799 | 5 | 76286134 | 0.10 | 1393 | intron |  |  |
| C-reactive protein | rs1130864 | 1 | 157949715 | 0.28 | 1401 | 3UTR |  |  |
| Chemokine ligand 12 | rs197452 | 10 | 44190246 | 0.16 | 6387 | intron |  |  |
|  | rs2297630 | 10 | 44191554 | 0.22 | 6387 | intron |  |  |
|  | rs266093 | 10 | 44186214 | 0.34 | 6387 | 3UTR |  |  |
|  | rs266094 | 10 | 44184306 | 0.13 | 6387 | flanking_3UTR |  |  |
|  | rs2839688 | 10 | 44195888 | 0.13 | 6387 | intron |  |  |
|  | rs2839695 | 10 | 44193855 | 0.16 | 6387 | 3UTR |  |  |
|  | rs7088876 | 10 | 44198647 | 0.16 | 6387 | intron |  |  |
| Chemokine ligand 17 | rs11671846 | 19 | 47632143 | 0.12 | 284340 | intron |  |  |
| Dimethylarginine dimethylaminohyrolase 1 | rs11161618 | 1 | 85687990 | 0.46 | 23576 | intron |  |  |
|  | rs1146383 | 1 | 85690917 | 0.18 | 23576 | intron |  |  |
|  | rs11587876 | 1 | 85687771 | 0.19 | 23576 | intron |  |  |
|  | rs12130380 | 1 | 85673897 | 0.20 | 23576 | intron |  |  |
|  | rs1241321 | 1 | 85700467 | 0.24 | 23576 | intron |  |  |
|  | rs13373844 | 1 | 85692347 | 0.31 | 23576 | intron |  |  |
|  | rs1498373 | 1 | 85563221 | 0.30 | 23576 | intron |  |  |
|  | rs17388521 | 1 | 85670492 | 0.16 | 23576 | intron |  |  |
|  | rs17590006 | 1 | 85561858 | 0.26 | 23576 | intron |  |  |
|  | rs2284800 | 1 | 85588198 | 0.04 | 23576 | intron |  |  |
|  | rs233112 | 1 | 85558339 | 0.36 | 23576 | 3UTR |  |  |
|  | rs233128 | 1 | 85567001 | 0.30 | 23576 | intron |  |  |
|  | rs480414 | 1 | 85700051 | 0.35 | 23576 | intron |  |  |
|  | rs527762 | 1 | 85701802 | 0.36 | 23576 | intron |  |  |
|  | rs600098 | 1 | 85685172 | 0.14 | 23576 | intron |  |  |
|  | rs6657732 | 1 | 85647083 | 0.40 | 23576 | intron |  |  |
|  | rs6672870 | 1 | 85664045 | 0.15 | 23576 | intron |  |  |
|  | rs669173 | 1 | 85672016 | 0.45 | 23576 | intron |  |  |
|  | rs877041 | 1 | 85696508 | 0.36 | 23576 | intron |  |  |
|  | rs974874 | 1 | 85697696 | 0.24 | 23576 | intron |  |  |
|  | rs997251 | 1 | 85617955 | 0.31 | 23576 | intron |  |  |
|  | rs986639 | 1 | 85836392 | 0.16 | 23576 | intron |  |  |
| Dimethylarginine dimethylaminohyrolase 2 | rs805305 | 6 | 31805366 | 0.34 | 23564 | intron |  |  |
|  | rs9267551 | 6 | 31805936 | 0.03 | 23564 | 5UTR |  |  |
| Dipeptidyl-peptidase 4 | rs12469968 | 2 | 162632163 | 0.42 | 1803 | intron |  |  |
|  | rs12995983 | 2 | 162608049 | 0.26 | 1803 | intron |  |  |
|  | rs2389643 | 2 | 162575238 | 0.05 | 1803 | intron |  |  |
|  | rs2909445 | 2 | 162563064 | 0.19 | 1803 | intron |  |  |
|  | rs2909448 | 2 | 162574501 | 0.42 | 1803 | intron |  |  |
|  | rs3788979 | 2 | 162609135 | 0.09 | 1803 | intron |  |  |
|  | rs4140685 | 2 | 162560325 | 0.35 | 1803 | intron |  |  |
|  | rs4664446 | 2 | 162618649 | 0.45 | 1803 | intron |  |  |
|  | rs6733162 | 2 | 162621744 | 0.35 | 1803 | intron |  |  |
|  | rs6741949 | 2 | 162618469 | 0.40 | 1803 | intron |  |  |
|  | rs741529 | 2 | 162627735 | 0.17 | 1803 | intron |  |  |
|  | rs873826 | 2 | 162626317 | 0.34 | 1803 | intron |  |  |
| Dopamine receptor D2 | rs1076563 | 11 | 112801119 | 0.36 | 1813 | intron |  |  |
|  | rs1079597 | 11 | 112801496 | 0.09 | 1813 | intron |  |  |
|  | rs11214613 | 11 | 112840469 | 0.06 | 1813 | intron |  |  |
|  | rs17529477 | 11 | 112822277 | 0.39 | 1813 | intron |  |  |
|  | rs2734841 | 11 | 112786986 | 0.38 | 1813 | intron |  |  |
|  | rs4245147 | 11 | 112823217 | 0.48 | 1813 | intron |  |  |
|  | rs4648317 | 11 | 112836742 | 0.16 | 1813 | intron |  |  |
|  | rs4648318 | 11 | 112818599 | 0.28 | 1813 | intron |  |  |
|  | rs7131056 | 11 | 112834984 | 0.31 | 1813 | intron |  |  |
| Endothelin 1 | rs1476046 | 6 | 12401207 | 0.22 | 1906 | intron |  |  |
|  | rs1626492 | 6 | 12403489 | 0.34 | 1906 | intron |  |  |
|  | rs1630736 | 6 | 12403973 | 0.41 | 1906 | intron |  |  |
|  | rs2071943 | 6 | 12403800 | 0.21 | 1906 | intron |  |  |
| Endothelin receptor type A | rs10008744 | 4 | 148664879 | 0.39 | 1909 | intron |  |  |
|  | rs1568136 | 4 | 148651890 | 0.33 | 1909 | intron |  |  |
|  | rs17612742 | 4 | 148634101 | 0.17 | 1909 | intron |  |  |
|  | rs1878404 | 4 | 148660075 | 0.31 | 1909 | intron |  |  |
|  | rs2048894 | 4 | 148671284 | 0.34 | 1909 | intron |  |  |
|  | rs2357924 | 4 | 148674915 | 0.20 | 1909 | intron |  |  |
|  | rs5335 | 4 | 148683290 | 0.45 | 1909 | 3UTR |  |  |
|  | rs6537484 | 4 | 148647343 | 0.46 | 1909 | intron |  |  |
|  | rs6537485 | 4 | 148647977 | 0.13 | 1909 | intron |  |  |
|  | rs6827096 | 4 | 148654896 | 0.20 | 1909 | intron |  |  |
|  | rs7655670 | 4 | 148643288 | 0.42 | 1909 | intron |  |  |
| Endothelin receptor type B | rs2329047 | 13 | 77399651 | 0.41 | 1910 | intron |  |  |
|  | rs3818416 | 13 | 77372469 | 0.33 | 1910 | intron |  |  |
|  | rs4884076 | 13 | 77439953 | 0.12 | 1910 | intron |  |  |
|  | rs4885492 | 13 | 77376306 | 0.14 | 1910 | intron |  |  |
|  | rs4885493 | 13 | 77381064 | 0.34 | 1910 | intron |  |  |
|  | rs7982910 | 13 | 77382132 | 0.36 | 1910 | intron |  |  |
| Ectonucleotide pyrophosphatase phosphodiesterase 1 | rs12201710 | 6 | 132184591 | 0.20 | 5167 | intron |  |  |
|  | rs1409181 | 6 | 132190993 | 0.46 | 5167 | intron |  |  |
|  | rs1830971 | 6 | 132190046 | 0.41 | 5167 | intron |  |  |
|  | rs6569759 | 6 | 132174809 | 0.43 | 5167 | intron |  |  |
|  | rs6916495 | 6 | 132201958 | 0.11 | 5167 | intron |  |  |
|  | rs6926970 | 6 | 132208983 | 0.21 | 5167 | intron |  |  |
|  | rs6939185 | 6 | 132180880 | 0.32 | 5167 | intron |  |  |
|  | rs703184 | 6 | 132196688 | 0.12 | 5167 | intron |  |  |
|  | rs7775386 | 6 | 132198842 | 0.12 | 5167 | intron |  |  |
|  | rs858338 | 6 | 132194900 | 0.22 | 5167 | intron |  |  |
|  | rs858339 | 6 | 132195590 | 0.32 | 5167 | intron |  |  |
|  | rs858341 | 6 | 132202148 | 0.43 | 5167 | intron |  |  |
|  | rs858343 | 6 | 132204140 | 0.30 | 5167 | intron |  |  |
|  | rs9375831 | 6 | 132206380 | 0.23 | 5167 | intron |  |  |
|  | rs9402349 | 6 | 132226801 | 0.13 | 5167 | intron |  |  |
|  | rs9493105 | 6 | 132180031 | 0.12 | 5167 | intron |  |  |
| Estrogen receptor 1 | rs11155813 | 6 | 152191128 | 0.12 | 2099 | intron |  |  |
|  | rs12154178 | 6 | 152292773 | 0.32 | 2099 | intron |  |  |
|  | rs12212176 | 6 | 152351700 | 0.14 | 2099 | intron |  |  |
|  | rs1709183 | 6 | 152235689 | 0.20 | 2099 | intron |  |  |
|  | rs1801132 | 6 | 152307215 | 0.25 | 2099 | coding |  |  |
|  | rs2144025 | 6 | 152349399 | 0.15 | 2099 | intron |  |  |
|  | rs2347867 | 6 | 152271543 | 0.30 | 2099 | intron |  |  |
|  | rs2982683 | 6 | 152340128 | 0.28 | 2099 | intron |  |  |
|  | rs2982712 | 6 | 152399872 | 0.41 | 2099 | intron |  |  |
|  | rs2982896 | 6 | 152441186 | 0.22 | 2099 | intron |  |  |
|  | rs3003925 | 6 | 152326151 | 0.19 | 2099 | intron |  |  |
|  | rs3020314 | 6 | 152312365 | 0.37 | 2099 | intron |  |  |
|  | rs3020317 | 6 | 152320434 | 0.22 | 2099 | intron |  |  |
|  | rs3020318 | 6 | 152331463 | 0.38 | 2099 | intron |  |  |
|  | rs3020364 | 6 | 152408811 | 0.36 | 2099 | intron |  |  |
|  | rs3020368 | 6 | 152412883 | 0.12 | 2099 | intron |  |  |
|  | rs3020383 | 6 | 152458472 | 0.11 | 2099 | intron |  |  |
|  | rs3020403 | 6 | 152337408 | 0.35 | 2099 | intron |  |  |
|  | rs3020407 | 6 | 152348954 | 0.32 | 2099 | intron |  |  |
|  | rs3020410 | 6 | 152308070 | 0.12 | 2099 | intron |  |  |
|  | rs3020434 | 6 | 152400633 | 0.18 | 2099 | intron |  |  |
|  | rs3798577 | 6 | 152462823 | 0.44 | 2099 | 3UTR |  |  |
|  | rs3853248 | 6 | 152181579 | 0.13 | 2099 | intron |  |  |
|  | rs532010 | 6 | 152172611 | 0.44 | 2099 | intron |  |  |
|  | rs6914211 | 6 | 152310233 | 0.14 | 2099 | intron |  |  |
|  | rs7757956 | 6 | 152358833 | 0.16 | 2099 | intron |  |  |
|  | rs827420 | 6 | 152219222 | 0.46 | 2099 | intron |  |  |
|  | rs827423 | 6 | 152197890 | 0.50 | 2099 | intron |  |  |
|  | rs926777 | 6 | 152346740 | 0.26 | 2099 | intron |  |  |
|  | rs9322335 | 6 | 152241822 | 0.18 | 2099 | intron |  |  |
|  | rs9322336 | 6 | 152242123 | 0.16 | 2099 | intron |  |  |
|  | rs9340835 | 6 | 152241624 | 0.32 | 2099 | intron |  |  |
|  | rs9340954 | 6 | 152361865 | 0.32 | 2099 | intron |  |  |
|  | rs9341052 | 6 | 152458318 | 0.05 | 2099 | intron |  |  |
|  | rs9397463 | 6 | 152346021 | 0.13 | 2099 | intron |  |  |
|  | rs9479130 | 6 | 152210149 | 0.46 | 2099 | intron |  |  |
|  | rs985192 | 6 | 152325171 | 0.22 | 2099 | intron |  |  |
|  | rs985694 | 6 | 152328318 | 0.17 | 2099 | intron |  |  |
|  | rs988328 | 6 | 152282843 | 0.16 | 2099 | intron |  |  |
| Coagulation factor VII | rs1475931 | 13 | 112811927 | 0.31 | 2155 | intron |  |  |
|  | rs488703 | 13 | 112818877 | 0.20 | 2155 | intron |  |  |
| Fatty acid binding protein 1, liver | rs1530273 | 2 | 88205822 | 0.25 | 2168 | intron |  |  |
|  | rs2197076 | 2 | 88203873 | 0.21 | 2168 | intron |  |  |
|  | rs2241883 | 2 | 88205181 | 0.29 | 2168 | coding |  |  |
|  | rs2919871 | 2 | 88207561 | 0.29 | 2168 | intron |  |  |
| TNF receptor superfamily, member 6 | rs10509561 | 10 | 90741892 | 0.29 | 355 | intron |  |  |
|  | rs1051070 | 10 | 90764752 | 0.08 | 355 | 3UTR |  |  |
|  | rs1468063 | 10 | 90765271 | 0.12 | 355 | 3UTR |  |  |
|  | rs1977389 | 10 | 90763474 | 0.43 | 355 | intron |  |  |
|  | rs2234978 | 10 | 90761809 | 0.27 | 355 | UTR |  |  |
|  | rs3740286 | 10 | 90741320 | 0.41 | 355 | intron |  |  |
|  | rs6586165 | 10 | 90745036 | 0.46 | 355 | intron |  |  |
|  | rs9658727 | 10 | 90751845 | 0.14 | 355 | intron |  |  |
|  | rs9658761 | 10 | 90759866 | 0.14 | 355 | intron |  |  |
|  | rs982764 | 10 | 90759978 | 0.30 | 355 | intron |  |  |
| Fibrillarin | rs12975624 | 19 | 45026877 | 0.49 | 2091 | intron |  |  |
|  | rs12983225 | 19 | 45027650 | 0.11 | 2091 | intron |  |  |
|  | rs3760924 | 19 | 45019152 | 0.17 | 2091 | intron |  |  |
|  | rs7250408 | 19 | 45025811 | 0.39 | 2091 | intron |  |  |
|  | rs7259470 | 19 | 45021318 | 0.29 | 2091 | intron |  |  |
| Fibrinogen α chain | rs2070016 | 4 | 155729764 | 0.22 | 2243 | intron |  |  |
|  | rs2070018 | 4 | 155728077 | 0.16 | 2243 | intron |  |  |
|  | rs2070022 | 4 | 155724398 | 0.15 | 2243 | 3UTR |  |  |
| Fibrinogen β chain | rs2227401 | 4 | 155705831 | 0.25 | 2244 | intron |  |  |
|  | rs2227412 | 4 | 155708545 | 0.12 | 2244 | intron |  |  |
| FMS-related tyrosine kinase 1 | rs12858139 | 13 | 27956068 | 0.38 | 2321 | intron |  |  |
|  | rs1324057 | 13 | 27823435 | 0.24 | 2321 | intron |  |  |
|  | rs1408245 | 13 | 27809820 | 0.15 | 2321 | intron |  |  |
|  | rs17086609 | 13 | 27827711 | 0.38 | 2321 | intron |  |  |
|  | rs17537653 | 13 | 27866510 | 0.14 | 2321 | intron |  |  |
|  | rs2256849 | 13 | 27943852 | 0.14 | 2321 | intron |  |  |
|  | rs2281827 | 13 | 27899721 | 0.30 | 2321 | intron |  |  |
|  | rs2296188 | 13 | 27791484 | 0.24 | 2321 | intron |  |  |
|  | rs2296189 | 13 | 27791642 | 0.26 | 2321 | coding |  |  |
|  | rs2296283 | 13 | 27861702 | 0.43 | 2321 | intron |  |  |
|  | rs2296285 | 13 | 27907673 | 0.30 | 2321 | intron |  |  |
|  | rs2387632 | 13 | 27814343 | 0.31 | 2321 | intron |  |  |
|  | rs3751395 | 13 | 27856955 | 0.47 | 2321 | intron |  |  |
|  | rs3751397 | 13 | 27858593 | 0.46 | 2321 | intron |  |  |
|  | rs3794399 | 13 | 27790892 | 0.11 | 2321 | intron |  |  |
|  | rs3794400 | 13 | 27800008 | 0.15 | 2321 | intron |  |  |
|  | rs4771249 | 13 | 27911414 | 0.28 | 2321 | intron |  |  |
|  | rs678714 | 13 | 27964057 | 0.10 | 2321 | intron |  |  |
|  | rs722503 | 13 | 27895052 | 0.32 | 2321 | intron |  |  |
|  | rs748252 | 13 | 27958502 | 0.34 | 2321 | intron |  |  |
|  | rs7987291 | 13 | 27792206 | 0.23 | 2321 | intron |  |  |
|  | rs7987649 | 13 | 27792415 | 0.28 | 2321 | intron |  |  |
|  | rs7992940 | 13 | 27921921 | 0.33 | 2321 | intron |  |  |
|  | rs7995976 | 13 | 27839060 | 0.21 | 2321 | intron |  |  |
|  | rs942364 | 13 | 27794097 | 0.09 | 2321 | intron |  |  |
|  | rs9508021 | 13 | 27865577 | 0.31 | 2321 | intron |  |  |
|  | rs9508026 | 13 | 27882400 | 0.28 | 2321 | intron |  |  |
|  | rs9513085 | 13 | 27818923 | 0.15 | 2321 | intron |  |  |
|  | rs9513088 | 13 | 27832707 | 0.30 | 2321 | intron |  |  |
|  | rs9551462 | 13 | 27851104 | 0.33 | 2321 | intron |  |  |
|  | rs9554314 | 13 | 27773789 | 0.09 | 2321 | flanking_3UTR |  |  |
|  | rs9554320 | 13 | 27784927 | 0.40 | 2321 | intron |  |  |
| Follistatin-like 3 | rs13730 | 19 | 633010 | 0.14 | 10272 | UTR |  |  |
| Fat mass and obesity associated protein | rs1421085 | 16 | 52358455 | 0.46 | 79068 | intron |  |  |
|  | rs17817449 | 16 | 52370868 | 0.44 | 79068 | intron |  |  |
| Glutamate decarboxylase 2 | rs11015008 | 10 | 26567596 | 0.25 | 2572 | intron |  |  |
|  | rs12243037 | 10 | 26564380 | 0.21 | 2572 | intron |  |  |
|  | rs1805398 | 10 | 26574815 | 0.08 | 2572 | intron |  |  |
|  | rs3781117 | 10 | 26552050 | 0.11 | 2572 | intron |  |  |
|  | rs4749107 | 10 | 26612848 | 0.31 | 2572 | intron |  |  |
|  | rs4994426 | 10 | 26605679 | 0.11 | 2572 | intron |  |  |
|  | rs7072137 | 10 | 26596973 | 0.09 | 2572 | intron |  |  |
|  | rs8190612 | 10 | 26552381 | 0.13 | 2572 | intron |  |  |
|  | rs928197 | 10 | 26629631 | 0.21 | 2572 | intron |  |  |
| Glucagon | rs5647 | 2 | 162713404 | 0.51 | 2641 | intron |  |  |
|  | rs7581952 | 2 | 162709102 | 0.50 | 2641 | intron |  |  |
| Glucokinase | rs2070971 | 7 | 44164108 | 0.11 | 2645 | intron |  |  |
|  | rs2268572 | 7 | 44161076 | 0.13 | 2645 | intron |  |  |
|  | rs2268575 | 7 | 44155799 | 0.25 | 2645 | intron |  |  |
|  | rs2268576 | 7 | 44155548 | 0.50 | 2645 | intron |  |  |
|  | rs2971676 | 7 | 44161007 | 0.07 | 2645 | intron |  |  |
| Growth differentiation factor 15 | rs1059369 | 19 | 18358141 | 0.22 | 9518 | coding |  |  |
|  | rs1059519 | 19 | 18358024 | 0.36 | 9518 | coding |  |  |
|  | rs1227731 | 19 | 18358903 | 0.17 | 9518 | intron |  |  |
| Glial cell-derived neurotrophic factor | rs10941370 | 5 | 37869176 | 0.43 | 2668 | intron |  |  |
|  | rs12521946 | 5 | 37860541 | 0.34 | 2668 | intron |  |  |
|  | rs1549250 | 5 | 37856978 | 0.41 | 2668 | intron |  |  |
|  | rs2910701 | 5 | 37865142 | 0.10 | 2668 | intron |  |  |
|  | rs2973041 | 5 | 37866083 | 0.15 | 2668 | intron |  |  |
|  | rs2973042 | 5 | 37863516 | 0.28 | 2668 | intron |  |  |
|  | rs2973043 | 5 | 37859157 | 0.24 | 2668 | intron |  |  |
|  | rs2973050 | 5 | 37853101 | 0.36 | 2668 | intron |  |  |
|  | rs3096140 | 5 | 37868590 | 0.30 | 2668 | intron |  |  |
|  | rs7731209 | 5 | 37852602 | 0.14 | 2668 | intron |  |  |
|  | rs884344 | 5 | 37859997 | 0.24 | 2668 | intron |  |  |
| Ghrelin | rs2075356 | 3 | 10303809 | 0.12 | 51738 | intron |  |  |
|  | rs26802 | 3 | 10307365 | 0.36 | 51738 | intron |  |  |
|  | rs35683 | 3 | 10303250 | 0.43 | 51738 | intron |  |  |
|  | rs42451 | 3 | 10305377 | 0.29 | 51738 | intron |  |  |
|  | rs4684677 | 3 | 10303453 | 0.04 | 51738 | coding |  |  |
| Growth hormone secretagogue receptor | rs572169 | 3 | 173648421 | 0.16 | 2693 | coding |  |  |
| Guanine nucleotide binding protein, β polypeptide 3 | rs5443 | 12 | 6825136 | 0.38 | 2784 | coding |  |  |
|  | rs5446 | 12 | 6826723 | 0.36 | 2784 | 3UTR |  |  |
| Glutathine S-transferase θ1 | rs2234735 | 22 | 24381652 | N/A | 2952 | intron |  |  |
| H19, imprinted maternally expressed transcript | rs2251375 | 11 | 1976072 | 0.31 | 283120 | flanking_5UTR |  |  |
| HNF1 homeobox A | rs1169286 | 12 | 119903439 | 0.43 | 6927 | intron |  |  |
|  | rs1169293 | 12 | 119910928 | 0.09 | 6927 | intron |  |  |
|  | rs1169300 | 12 | 119915608 | 0.33 | 6927 | intron |  |  |
|  | rs1169302 | 12 | 119916685 | 0.48 | 6927 | intron |  |  |
|  | rs1169307 | 12 | 119922765 | 0.32 | 6927 | intron |  |  |
|  | rs12427353 | 12 | 119911284 | 0.09 | 6927 | intron |  |  |
|  | rs2259816 | 12 | 119919970 | 0.37 | 6927 | intron |  |  |
|  | rs2393791 | 12 | 119908339 | 0.39 | 6927 | intron |  |  |
| HNF1 homeobox B | rs1016990 | 17 | 33163028 | 0.27 | 6928 | 3UTR |  |  |
|  | rs10962 | 17 | 33120564 | 0.24 | 6928 | 3UTR |  |  |
|  | rs11656817 | 17 | 33131012 | 0.07 | 6928 | intron |  |  |
|  | rs11868513 | 17 | 33126805 | 0.17 | 6928 | intron |  |  |
|  | rs2074428 | 17 | 33135772 | 0.48 | 6928 | intron |  |  |
|  | rs2074430 | 17 | 33134628 | 0.26 | 6928 | intron |  |  |
|  | rs2107131 | 17 | 33160802 | 0.33 | 6928 | intron |  |  |
|  | rs2158254 | 17 | 33139608 | 0.48 | 6928 | intron |  |  |
|  | rs2189303 | 17 | 33134218 | 0.24 | 6928 | intron |  |  |
|  | rs2285740 | 17 | 33142841 | 0.40 | 6928 | intron |  |  |
|  | rs3094508 | 17 | 33137048 | 0.29 | 6928 | intron |  |  |
|  | rs3110640 | 17 | 33122936 | 0.39 | 6928 | intron |  |  |
|  | rs3110641 | 17 | 33121530 | 0.33 | 6928 | intron |  |  |
|  | rs3110644 | 17 | 33147309 | 0.11 | 6928 | intron |  |  |
|  | rs3744763 | 17 | 33164998 | 0.35 | 6928 | 3UTR |  |  |
|  | rs3786127 | 17 | 36087874 | 0.11 | 6928 | intron |  |  |
|  | rs4430796 | 17 | 33172153 | 0.47 | 6928 | intron |  |  |
|  | rs4794758 | 17 | 33154541 | 0.34 | 6928 | intron |  |  |
|  | rs7405696 | 17 | 33176148 | 0.41 | 6928 | intron |  |  |
|  | rs7407025 | 17 | 33154923 | 0.26 | 6928 | intron |  |  |
|  | rs7501939 | 17 | 33175269 | 0.39 | 6928 | intron |  |  |
|  | rs8075185 | 17 | 33134329 | N/A | 6928 | intron |  |  |
|  | rs9892543 | 17 | 33142072 | 0.25 | 6928 | intron |  |  |
| Hepatocyte nuclear factor 4α | rs11574736 | 20 | 42472951 | 0.21 | 3172 | intron |  |  |
|  | rs11574738 | 20 | 42475994 | 0.21 | 3172 | intron |  |  |
|  | rs3212183 | 20 | 42468552 | 0.41 | 3172 | intron |  |  |
|  | rs3212198 | 20 | 42477776 | 0.34 | 3172 | intron |  |  |
|  | rs6093976 | 20 | 42469194 | 0.22 | 3172 | intron |  |  |
|  | rs6093978 | 20 | 42475005 | 0.40 | 3172 | intron |  |  |
|  | rs745975 | 20 | 42468107 | 0.24 | 3172 | intron |  |  |
|  | rs8114057 | 20 | 42469866 | 0.46 | 3172 | intron |  |  |
|  | rs8116574 | 20 | 42464223 | 0.18 | 3172 | intron |  |  |
| 11β dehydrogenase 1 | rs2235543 | 1 | 207927291 | 0.15 | 3290 | intron |  |  |
|  | rs3753519 | 1 | 207942138 | 0.09 | 3290 | intron |  |  |
|  | rs3766619 | 1 | 207961921 | 0.17 | 3290 | intron |  |  |
|  | rs4844880 | 1 | 207937539 | 0.16 | 3290 | intron |  |  |
|  | rs6672256 | 1 | 207960666 | 0.17 | 3290 | intron |  |  |
| Heat shock 70kDa protein 1A | rs1043618 | 6 | 31891486 | 0.37 | 3303 | 5UTR |  |  |
| Serotonin receptor 2A | rs1002513 | 13 | 46352441 | 0.09 | 3356 | intron |  |  |
|  | rs12584920 | 13 | 46363038 | 0.11 | 3356 | intron |  |  |
|  | rs1328684 | 13 | 46364231 | 0.31 | 3356 | intron |  |  |
|  | rs1745837 | 13 | 46322813 | 0.32 | 3356 | intron |  |  |
|  | rs1923882 | 13 | 46309662 | 0.25 | 3356 | intron |  |  |
|  | rs1923886 | 13 | 46321292 | 0.37 | 3356 | intron |  |  |
|  | rs1928042 | 13 | 46335217 | 0.16 | 3356 | intron |  |  |
|  | rs2070037 | 13 | 46365071 | 0.15 | 3356 | intron |  |  |
|  | rs2224721 | 13 | 46330155 | 0.30 | 3356 | intron |  |  |
|  | rs2296973 | 13 | 46364782 | 0.24 | 3356 | intron |  |  |
|  | rs2770296 | 13 | 46338561 | 0.24 | 3356 | intron |  |  |
|  | rs3125 | 13 | 46306852 | 0.13 | 3356 | 3UTR |  |  |
|  | rs582854 | 13 | 46343878 | 0.28 | 3356 | intron |  |  |
|  | rs6561335 | 13 | 46331498 | 0.20 | 3356 | intron |  |  |
|  | rs731779 | 13 | 46350039 | 0.11 | 3356 | intron |  |  |
|  | rs7330636 | 13 | 46321593 | 0.45 | 3356 | intron |  |  |
|  | rs7984966 | 13 | 46327447 | 0.31 | 3356 | intron |  |  |
|  | rs7997012 | 13 | 46309986 | 0.29 | 3356 | intron |  |  |
|  | rs927544 | 13 | 46354052 | 0.23 | 3356 | intron |  |  |
|  | rs9316232 | 13 | 46324723 | 0.34 | 3356 | intron |  |  |
|  | rs9316233 | 13 | 46331356 | 0.28 | 3356 | intron |  |  |
|  | rs9526240 | 13 | 46329472 | 0.16 | 3356 | intron |  |  |
|  | rs9526245 | 13 | 46343968 | 0.20 | 3356 | intron |  |  |
|  | rs9534493 | 13 | 46316384 | 0.14 | 3356 | intron |  |  |
|  | rs9534495 | 13 | 46327229 | 0.41 | 3356 | intron |  |  |
|  | rs9534505 | 13 | 46358745 | 0.12 | 3356 | intron |  |  |
|  | rs9534511 | 13 | 46366581 | 0.34 | 3356 | intron |  |  |
|  | rs977003 | 13 | 46313002 | 0.44 | 3356 | intron |  |  |
|  | rs985933 | 13 | 46353864 | 0.38 | 3356 | intron |  |  |
|  | rs2274639 | 13 | 47430263 | 0.18 | 3356 | intron |  |  |
| Insulin-like growth factor 1 | rs1019731 | 12 | 101388555 | 0.10 | 3479 | intron |  |  |
|  | rs10735380 | 12 | 101368366 | 0.29 | 3479 | intron |  |  |
|  | rs12821878 | 12 | 101391797 | 0.25 | 3479 | intron |  |  |
|  | rs1520220 | 12 | 101320652 | 0.26 | 3479 | intron |  |  |
|  | rs17727841 | 12 | 101333760 | 0.18 | 3479 | intron |  |  |
|  | rs2162679 | 12 | 101395389 | 0.17 | 3479 | intron |  |  |
|  | rs5742632 | 12 | 101380604 | 0.23 | 3479 | intron |  |  |
|  | rs5742667 | 12 | 101347608 | 0.28 | 3479 | intron |  |  |
|  | rs6214 | 12 | 101317699 | 0.37 | 3479 | 3UTR |  |  |
|  | rs7136446 | 12 | 101362645 | 0.40 | 3479 | intron |  |  |
|  | rs7956547 | 12 | 101382946 | 0.24 | 3479 | intron |  |  |
| Insulin-like growth factor 1 receptor | rs11247361 | 15 | 97024915 | 0.34 | 3480 | intron |  |  |
|  | rs11247372 | 15 | 97134201 | 0.21 | 3480 | intron |  |  |
|  | rs11630259 | 15 | 97248471 | 0.36 | 3480 | intron |  |  |
|  | rs12439656 | 15 | 97270465 | 0.16 | 3480 | intron |  |  |
|  | rs12442093 | 15 | 97283409 | 0.15 | 3480 | intron |  |  |
|  | rs12591122 | 15 | 97315935 | 0.17 | 3480 | intron |  |  |
|  | rs12901570 | 15 | 97071145 | 0.06 | 3480 | intron |  |  |
|  | rs12908948 | 15 | 97149695 | 0.07 | 3480 | intron |  |  |
|  | rs12916884 | 15 | 97305411 | 0.24 | 3480 | intron |  |  |
|  | rs1319869 | 15 | 97030008 | 0.17 | 3480 | intron |  |  |
|  | rs1879612 | 15 | 97216231 | 0.42 | 3480 | intron |  |  |
|  | rs2137680 | 15 | 97130264 | 0.33 | 3480 | intron |  |  |
|  | rs2229765 | 15 | 97295748 | 0.43 | 3480 | coding |  |  |
|  | rs2684777 | 15 | 97214217 | 0.24 | 3480 | intron |  |  |
|  | rs2684780 | 15 | 97224402 | 0.24 | 3480 | intron |  |  |
|  | rs2684781 | 15 | 97227071 | 0.22 | 3480 | intron |  |  |
|  | rs2684790 | 15 | 97309063 | 0.16 | 3480 | intron |  |  |
|  | rs2684792 | 15 | 97305604 | 0.42 | 3480 | intron |  |  |
|  | rs2684794 | 15 | 97302476 | 0.08 | 3480 | intron |  |  |
|  | rs2684796 | 15 | 97300915 | 0.13 | 3480 | intron |  |  |
|  | rs2684799 | 15 | 97299355 | 0.39 | 3480 | intron |  |  |
|  | rs2684800 | 15 | 97298293 | 0.36 | 3480 | intron |  |  |
|  | rs2684805 | 15 | 97283608 | 0.07 | 3480 | intron |  |  |
|  | rs2684806 | 15 | 97282808 | 0.50 | 3480 | intron |  |  |
|  | rs2684807 | 15 | 97281977 | 0.47 | 3480 | intron |  |  |
|  | rs2684808 | 15 | 97276241 | 0.30 | 3480 | intron |  |  |
|  | rs2684810 | 15 | 97271768 | 0.27 | 3480 | intron |  |  |
|  | rs2684811 | 15 | 97271086 | 0.19 | 3480 | intron |  |  |
|  | rs2715419 | 15 | 97302243 | 0.44 | 3480 | intron |  |  |
|  | rs2871865 | 15 | 97012419 | 0.17 | 3480 | intron |  |  |
|  | rs2871974 | 15 | 97101597 | 0.43 | 3480 | intron |  |  |
|  | rs3743253 | 15 | 97317597 | 0.15 | 3480 | intron |  |  |
|  | rs3743259 | 15 | 97260684 | 0.26 | 3480 | intron |  |  |
|  | rs4246340 | 15 | 97184128 | 0.38 | 3480 | intron |  |  |
|  | rs4965438 | 15 | 97288952 | 0.23 | 3480 | intron |  |  |
|  | rs4966011 | 15 | 97021529 | 0.36 | 3480 | intron |  |  |
|  | rs4966012 | 15 | 97043671 | 0.29 | 3480 | intron |  |  |
|  | rs4966013 | 15 | 97062907 | 0.29 | 3480 | intron |  |  |
|  | rs4966015 | 15 | 97065655 | 0.15 | 3480 | intron |  |  |
|  | rs4966039 | 15 | 97275338 | 0.21 | 3480 | intron |  |  |
|  | rs4966043 | 15 | 97281658 | 0.34 | 3480 | intron |  |  |
|  | rs4966044 | 15 | 97283721 | 0.36 | 3480 | intron |  |  |
|  | rs4966046 | 15 | 97292798 | 0.15 | 3480 | intron |  |  |
|  | rs6598554 | 15 | 97172789 | 0.49 | 3480 | intron |  |  |
|  | rs7162314 | 15 | 97307439 | 0.22 | 3480 | intron |  |  |
|  | rs7166348 | 15 | 97065318 | 0.21 | 3480 | intron |  |  |
|  | rs7168671 | 15 | 97272485 | 0.31 | 3480 | intron |  |  |
|  | rs7169544 | 15 | 97275275 | 0.40 | 3480 | intron |  |  |
|  | rs7175052 | 15 | 97125473 | 0.26 | 3480 | intron |  |  |
|  | rs8026157 | 15 | 97313910 | 0.36 | 3480 | intron |  |  |
|  | rs8027457 | 15 | 97021624 | 0.47 | 3480 | intron |  |  |
|  | rs8027767 | 15 | 97115026 | 0.46 | 3480 | intron |  |  |
|  | rs8030950 | 15 | 97221544 | 0.31 | 3480 | intron |  |  |
|  | rs8032477 | 15 | 97072077 | 0.48 | 3480 | intron |  |  |
|  | rs8034284 | 15 | 97313771 | 0.43 | 3480 | intron |  |  |
|  | rs8035410 | 15 | 97213357 | 0.04 | 3480 | intron |  |  |
|  | rs871335 | 15 | 97297469 | 0.24 | 3480 | intron |  |  |
|  | rs883149 | 15 | 97267224 | 0.18 | 3480 | intron |  |  |
|  | rs907801 | 15 | 97139270 | 0.16 | 3480 | intron |  |  |
|  | rs939626 | 15 | 97310699 | 0.45 | 3480 | intron |  |  |
|  | rs9672254 | 15 | 97315608 | 0.42 | 3480 | intron |  |  |
|  | rs9920651 | 15 | 97280163 | 0.15 | 3480 | intron |  |  |
| Insulin-like growth factor 2 | rs3213216 | 11 | 2114755 | 0.38 | 3481 | intron |  |  |
|  | rs3213223 | 11 | 2113506 | 0.14 | 3481 | intron |  |  |
|  | rs3213225 | 11 | 2113112 | 0.49 | 3481 | intron |  |  |
|  | rs680 | 11 | 2110210 | 0.34 | 3481 | intron |  |  |
|  | rs734351 | 11 | 2112789 | 0.48 | 3481 | intron |  |  |
| Insulin-like growth factor 2 receptor | rs1003737 | 6 | 160350646 | 0.40 | 3482 | intron |  |  |
|  | rs1867348 | 6 | 160376153 | 0.06 | 3482 | intron |  |  |
|  | rs2065396 | 6 | 160409734 | 0.41 | 3482 | intron |  |  |
|  | rs2274849 | 6 | 160399872 | 0.18 | 3482 | intron |  |  |
|  | rs2277070 | 6 | 160365783 | 0.13 | 3482 | intron |  |  |
|  | rs2297370 | 6 | 160444763 | 0.17 | 3482 | intron |  |  |
|  | rs3734181 | 6 | 160349873 | 0.19 | 3482 | intron |  |  |
|  | rs3777406 | 6 | 160416045 | 0.17 | 3482 | intron |  |  |
|  | rs3777411 | 6 | 160396935 | 0.24 | 3482 | intron |  |  |
|  | rs3777421 | 6 | 160313376 | 0.29 | 3482 | intron |  |  |
|  | rs3798189 | 6 | 160355559 | 0.47 | 3482 | intron |  |  |
|  | rs3798197 | 6 | 160325079 | 0.26 | 3482 | intron |  |  |
|  | rs3798207 | 6 | 160314237 | 0.09 | 3482 | intron |  |  |
|  | rs3798209 | 6 | 160313860 | 0.18 | 3482 | intron |  |  |
|  | rs3822845 | 6 | 160330488 | 0.10 | 3482 | intron |  |  |
|  | rs384167 | 6 | 160396257 | 0.09 | 3482 | intron |  |  |
|  | rs416572 | 6 | 160387880 | 0.23 | 3482 | intron |  |  |
|  | rs435612 | 6 | 160383926 | 0.10 | 3482 | intron |  |  |
|  | rs4709391 | 6 | 160378747 | 0.19 | 3482 | intron |  |  |
|  | rs4709395 | 6 | 160398779 | 0.34 | 3482 | intron |  |  |
|  | rs600324 | 6 | 160439266 | 0.06 | 3482 | intron |  |  |
|  | rs609207 | 6 | 160385743 | 0.38 | 3482 | intron |  |  |
|  | rs635551 | 6 | 160324822 | 0.14 | 3482 | intron |  |  |
|  | rs6455680 | 6 | 160367978 | 0.34 | 3482 | intron |  |  |
|  | rs648253 | 6 | 160389139 | 0.49 | 3482 | intron |  |  |
|  | rs687088 | 6 | 160385044 | 0.29 | 3482 | intron |  |  |
|  | rs688359 | 6 | 160385281 | 0.38 | 3482 | intron |  |  |
|  | rs6903540 | 6 | 160330321 | 0.14 | 3482 | intron |  |  |
|  | rs6917747 | 6 | 160322695 | 0.15 | 3482 | intron |  |  |
|  | rs761388 | 6 | 160321306 | 0.08 | 3482 | intron |  |  |
|  | rs8191711 | 6 | 160333064 | 0.12 | 3482 | intron |  |  |
|  | rs8191745 | 6 | 160352321 | 0.17 | 3482 | intron |  |  |
|  | rs8191772 | 6 | 160381324 | 0.31 | 3482 | intron |  |  |
|  | rs998075 | 6 | 160388268 | 0.48 | 3482 | coding |  |  |
| Insulin-like growth factor binding protein 1 | rs1874479 | 7 | 45898751 | 0.13 | 3484 | intron |  |  |
|  | rs4619 | 7 | 45899194 | 0.27 | 3484 | coding |  |  |
| Insulin-like growth factor binding protein 3 | rs2453839 | 7 | 45920098 | 0.22 | 3486 | intron |  |  |
|  | rs2471551 | 7 | 45923580 | 0.29 | 3486 | intron |  |  |
|  | rs3110697 | 7 | 45921554 | 0.43 | 3486 | intron |  |  |
|  | rs6670 | 7 | 45918779 | 0.27 | 3486 | 3UTR |  |  |
| Interleukin 18 | rs1834481 | 11 | 111529037 | 0.12 | 3606 | intron |  |  |
|  | rs360722 | 11 | 111531913 | 0.17 | 3606 | intron |  |  |
|  | rs3882891 | 11 | 111519971 | 0.44 | 3606 | intron |  |  |
|  | rs549908 | 11 | 111526126 | 0.27 | 3606 | coding |  |  |
|  | rs5744280 | 11 | 111521724 | 0.42 | 3606 | intron |  |  |
| Interleukin 18 receptor 1 | rs11465596 | 2 | 102353525 | 0.09 | 8809 | intron |  |  |
|  | rs1362348 | 2 | 102351056 | 0.37 | 8809 | intron |  |  |
|  | rs2080289 | 2 | 102361452 | 0.18 | 8809 | intron |  |  |
|  | rs2287033 | 2 | 102377669 | 0.43 | 8809 | intron |  |  |
|  | rs3732127 | 2 | 102380182 | 0.22 | 8809 | 3UTR |  |  |
|  | rs3771170 | 2 | 102352412 | 0.24 | 8809 | intron |  |  |
|  | rs7558013 | 2 | 102359238 | 0.15 | 8809 | intron |  |  |
|  | rs7579737 | 2 | 102353793 | 0.39 | 8809 | intron |  |  |
| Interleukin 1α | rs2856836 | 2 | 113248554 | 0.37 | 3552 | 3UTR |  |  |
|  | rs2856838 | 2 | 113256443 | 0.32 | 3552 | intron |  |  |
|  | rs3783546 | 2 | 113251301 | 0.26 | 3552 | intron |  |  |
| Interleukin 1β | rs1143634 | 2 | 113306861 | 0.31 | 3553 | coding |  |  |
| Interleukin 1 receptor 2 | rs2072474 | 2 | 102005641 | 0.19 | 7850 | intron |  |  |
|  | rs2072476 | 2 | 102003296 | 0.14 | 7850 | intron |  |  |
|  | rs2110562 | 2 | 101996072 | 0.47 | 7850 | intron |  |  |
|  | rs3218979 | 2 | 102008559 | 0.15 | 7850 | intron |  |  |
|  | rs3218984 | 2 | 102010893 | 0.22 | 7850 | intron |  |  |
|  | rs4851522 | 2 | 101977260 | 0.17 | 7850 | intron |  |  |
|  | rs4851526 | 2 | 101984802 | 0.38 | 7850 | intron |  |  |
| Interleukin 6 | rs1554606 | 7 | 22735232 | 0.28 | 3569 | intron |  |  |
|  | rs2069840 | 7 | 22735097 | 0.26 | 3569 | intron |  |  |
| Interleukin 6 receptor | rs1386821 | 1 | 152648673 | 0.13 | 3570 | intron |  |  |
|  | rs2229238 | 1 | 152704520 | 0.18 | 3570 | 3UTR |  |  |
|  | rs4075015 | 1 | 152655820 | 0.50 | 3570 | intron |  |  |
|  | rs4240872 | 1 | 152702819 | 0.23 | 3570 | intron |  |  |
|  | rs4553185 | 1 | 152677579 | 0.45 | 3570 | intron |  |  |
|  | rs4845374 | 1 | 152693571 | 0.17 | 3570 | intron |  |  |
|  | rs4845623 | 1 | 152682401 | 0.39 | 3570 | intron |  |  |
| Inhibin βA | rs11770488 | 7 | 41699379 | 0.07 | 3624 | intron |  |  |
|  | rs2237432 | 7 | 41701559 | 0.27 | 3624 | intron |  |  |
|  | rs2237436 | 7 | 41697456 | 0.40 | 3624 | intron |  |  |
|  | rs3801158 | 7 | 41705666 | 0.19 | 3624 | intron |  |  |
| Insulin | rs3842748 | 11 | 2137971 | 0.17 | 3630 | intron |  |  |
|  | rs3842756 | 11 | 2137348 | 0.15 | 3630 | flanking_3UTR |  |  |
| Insulin-induced gene 2 | rs12464355 | 2 | 118566320 | 0.09 | 51141 | intron |  |  |
|  | rs2042492 | 2 | 118571394 | 0.24 | 51141 | intron |  |  |
|  | rs2161829 | 2 | 118573384 | 0.38 | 51141 | intron |  |  |
|  | rs7566605 | 2 | 118552495 | 0.34 | 51141 | flanking_5UTR |  |  |
|  | rs889904 | 2 | 118576941 | 0.50 | 51141 | intron |  |  |
|  | rs9308762 | 2 | 118580344 | 0.14 | 51141 | intron |  |  |
| Insulin receptor | rs1035942 | 19 | 7150803 | 0.27 | 3643 | intron |  |  |
|  | rs10401628 | 19 | 7077218 | 0.07 | 3643 | intron |  |  |
|  | rs10414819 | 19 | 7202702 | 0.32 | 3643 | intron |  |  |
|  | rs10416429 | 19 | 7181438 | 0.32 | 3643 | intron |  |  |
|  | rs10417205 | 19 | 7205707 | 0.35 | 3643 | intron |  |  |
|  | rs10426094 | 19 | 7156240 | 0.21 | 3643 | intron |  |  |
|  | rs10500204 | 19 | 7133963 | 0.38 | 3643 | intron |  |  |
|  | rs1051690 | 19 | 7067963 | 0.11 | 3643 | 3UTR |  |  |
|  | rs11667110 | 19 | 7087609 | 0.30 | 3643 | intron |  |  |
|  | rs11672739 | 19 | 7085266 | 0.08 | 3643 | intron |  |  |
|  | rs12459488 | 19 | 7157073 | 0.27 | 3643 | intron |  |  |
|  | rs12460755 | 19 | 7182679 | 0.30 | 3643 | intron |  |  |
|  | rs12971499 | 19 | 7165282 | 0.42 | 3643 | intron |  |  |
|  | rs12979424 | 19 | 7224492 | 0.31 | 3643 | intron |  |  |
|  | rs1366234 | 19 | 7118817 | 0.30 | 3643 | intron |  |  |
|  | rs17175860 | 19 | 7186146 | 0.18 | 3643 | intron |  |  |
|  | rs17253937 | 19 | 7135801 | 0.17 | 3643 | intron |  |  |
|  | rs17254521 | 19 | 7189695 | 0.50 | 3643 | intron |  |  |
|  | rs2059807 | 19 | 7117109 | 0.34 | 3643 | intron |  |  |
|  | rs2115386 | 19 | 7147565 | 0.48 | 3643 | intron |  |  |
|  | rs2860173 | 19 | 7080086 | 0.05 | 3643 | intron |  |  |
|  | rs2860175 | 19 | 7083081 | 0.16 | 3643 | intron |  |  |
|  | rs2860184 | 19 | 7238748 | 0.39 | 3643 | intron |  |  |
|  | rs3745545 | 19 | 7162841 | 0.18 | 3643 | intron |  |  |
|  | rs3852876 | 19 | 7190555 | 0.31 | 3643 | intron |  |  |
|  | rs4247374 | 19 | 7203756 | 0.10 | 3643 | intron |  |  |
|  | rs4499341 | 19 | 7151990 | 0.38 | 3643 | intron |  |  |
|  | rs4804195 | 19 | 7205944 | 0.39 | 3643 | intron |  |  |
|  | rs4804377 | 19 | 7145976 | 0.31 | 3643 | intron |  |  |
|  | rs4804404 | 19 | 7169382 | 0.22 | 3643 | intron |  |  |
|  | rs4804416 | 19 | 7174848 | 0.38 | 3643 | intron |  |  |
|  | rs6510950 | 19 | 7087489 | 0.08 | 3643 | intron |  |  |
|  | rs6510959 | 19 | 7135238 | 0.18 | 3643 | intron |  |  |
|  | rs6510960 | 19 | 7154732 | 0.10 | 3643 | intron |  |  |
|  | rs6510976 | 19 | 7217944 | 0.45 | 3643 | intron |  |  |
|  | rs7254487 | 19 | 7220321 | 0.27 | 3643 | intron |  |  |
|  | rs7254921 | 19 | 7229452 | 0.50 | 3643 | intron |  |  |
|  | rs7508679 | 19 | 7173832 | 0.44 | 3643 | intron |  |  |
|  | rs8103483 | 19 | 7096374 | 0.46 | 3643 | intron |  |  |
|  | rs8109559 | 19 | 7122629 | 0.24 | 3643 | intron |  |  |
|  | rs8110116 | 19 | 7094711 | 0.17 | 3643 | intron |  |  |
|  | rs8111710 | 19 | 7243583 | 0.20 | 3643 | intron |  |  |
|  | rs8112883 | 19 | 7130320 | 0.31 | 3643 | intron |  |  |
|  | rs890860 | 19 | 7179165 | 0.19 | 3643 | intron |  |  |
|  | rs919275 | 19 | 7212441 | 0.48 | 3643 | intron |  |  |
| Insulin receptor substrate 1 | rs17208470 | 2 | 227312149 | 0.08 | 3667 | flanking_5UTR |  |  |
| Insulin receptor substrate 2 | rs11618950 | 13 | 109232311 | 0.15 | 8660 | intron |  |  |
|  | rs2241745 | 13 | 109220532 | 0.12 | 8660 | intron |  |  |
|  | rs2289046 | 13 | 109205907 | 0.30 | 8660 | 3UTR |  |  |
|  | rs4771644 | 13 | 109211818 | 0.47 | 8660 | intron |  |  |
|  | rs4771645 | 13 | 109225129 | 0.39 | 8660 | intron |  |  |
|  | rs4773088 | 13 | 109219885 | 0.40 | 8660 | intron |  |  |
|  | rs7323191 | 13 | 109222076 | 0.13 | 8660 | intron |  |  |
|  | rs7997595 | 13 | 109228769 | 0.15 | 8660 | intron |  |  |
|  | rs7999797 | 13 | 109224001 | 0.44 | 8660 | intron |  |  |
|  | rs913949 | 13 | 109209797 | 0.14 | 8660 | intron |  |  |
| Kallikrein 1 | rs2659058 | 19 | 56017918 | 0.33 | 3816 | intron |  |  |
|  | rs2740502 | 19 | 56017998 | 0.41 | 3816 | intron |  |  |
|  | rs5517 | 19 | 56015044 | 0.26 | 3816 | coding |  |  |
| Leptin | rs11760956 | 7 | 127678323 | 0.33 | 3952 | intron |  |  |
|  | rs2278815 | 7 | 127669087 | 0.43 | 3952 | intron |  |  |
|  | rs3828942 | 7 | 127681541 | 0.45 | 3952 | intron |  |  |
| Leptin receptor | rs1137100 | 1 | 65809029 | 0.15 | 3953 | coding |  |  |
|  | rs11585329 | 1 | 65846402 | 0.15 | 3953 | intron |  |  |
|  | rs1171265 | 1 | 65775840 | 0.28 | 3953 | flanking_5UTR |  |  |
|  | rs1171274 | 1 | 65753426 | 0.16 | 3953 | flanking_5UTR |  |  |
|  | rs1171276 | 1 | 65760037 | 0.29 | 3953 | flanking_5UTR |  |  |
|  | rs1475397 | 1 | 65755746 | 0.42 | 3953 | flanking_5UTR |  |  |
|  | rs1938484 | 1 | 65853870 | 0.07 | 3953 | intron |  |  |
|  | rs4606347 | 1 | 65845949 | 0.24 | 3953 | intron |  |  |
|  | rs4655537 | 1 | 65831389 | 0.38 | 3953 | intron |  |  |
|  | rs4655539 | 1 | 65832251 | 0.36 | 3953 | intron |  |  |
|  | rs6673324 | 1 | 65803651 | 0.40 | 3953 | flanking_5UTR |  |  |
|  | rs7535099 | 1 | 65823585 | 0.22 | 3953 | intron |  |  |
|  | rs12145690 | 1 | 65659601 | 0.45 | 54741 | intron |  |  |
|  | rs17127618 | 1 | 65667082 | 0.14 | 54741 | intron |  |  |
|  | rs17412175 | 1 | 65677474 | 0.24 | 54741 | flanking_3UTR |  |  |
|  | rs3790433 | 1 | 65666930 | 0.34 | 54741 | intron |  |  |
|  | rs4655802 | 1 | 65660819 | 0.33 | 54741 | intron |  |  |
|  | rs6704167 | 1 | 65710468 | 0.27 | 54741 | flanking_3UTR |  |  |
|  | rs7602 | 1 | 65670539 | 0.21 | 54741 | 3UTR |  |  |
|  | rs9436297 | 1 | 65661442 | 0.10 | 54741 | intron |  |  |
|  | rs9436299 | 1 | 65665476 | 0.42 | 54741 | intron |  |  |
|  | rs9436301 | 1 | 65668515 | 0.22 | 54741 | intron |  |  |
|  | rs9436739 | 1 | 65663287 | 0.10 | 54741 | intron |  |  |
|  | rs970467 | 1 | 65679350 | 0.14 | 54741 | flanking_3UTR |  |  |
|  | rs970468 | 1 | 65679078 | 0.44 | 54741 | flanking_3UTR |  |  |
| Leprecan-like 2 | rs1047771 | 12 | 6811282 | 0.32 | 10536 | coding |  |  |
|  | rs1129649 | 12 | 6818729 | 0.34 | 10536 | coding |  |  |
|  | rs2269355 | 12 | 6816175 | 0.49 | 10536 | intron |  |  |
|  | rs3741920 | 12 | 6809133 | 0.33 | 10536 | intron |  |  |
|  | rs3759347 | 12 | 6817941 | 0.31 | 10536 | intron |  |  |
|  | rs4963511 | 12 | 6813631 | 0.07 | 10536 | intron |  |  |
|  | rs4963517 | 12 | 6818061 | 0.45 | 10536 | intron |  |  |
|  | rs5439 | 12 | 6948882 | 0.10 | 10536 | coding |  |  |
| Hormone-sensitive lipase | rs10417560 | 19 | 47614792 | 0.02 | 3991 | intron |  |  |
|  | rs10423978 | 19 | 47593270 | 0.51 | 3991 | flanking_3UTR |  |  |
| N/A | rs501120 | 10 | 44073873 | 0.28 | 728347 | flanking_3UTR |  |  |
| N/A | rs1333049 | 9 | 22115503 | 0.43 | 729983 | flanking_5UTR |  |  |
| Lipoprotein lipase | rs10099160 | 8 | 19866095 | 0.13 | 4023 | intron |  |  |
|  | rs1059611 | 8 | 19868843 | 0.13 | 4023 | 3UTR |  |  |
|  | rs1534649 | 8 | 19843921 | 0.39 | 4023 | intron |  |  |
|  | rs17410577 | 8 | 19843825 | 0.16 | 4023 | intron |  |  |
|  | rs253 | 8 | 19855697 | 0.41 | 4023 | intron |  |  |
|  | rs264 | 8 | 19857460 | 0.16 | 4023 | intron |  |  |
|  | rs270 | 8 | 19857956 | 0.10 | 4023 | intron |  |  |
|  | rs283 | 8 | 19859378 | 0.19 | 4023 | intron |  |  |
|  | rs285 | 8 | 19859469 | 0.44 | 4023 | intron |  |  |
|  | rs327 | 8 | 19863816 | 0.32 | 4023 | intron |  |  |
|  | rs4922115 | 8 | 19867110 | 0.18 | 4023 | 3UTR |  |  |
| Low density lipoprotein receptor-related protein 8 | rs12035926 | 1 | 53524722 | 0.29 | 7804 | intron |  |  |
|  | rs12037959 | 1 | 53540539 | 0.19 | 7804 | intron |  |  |
|  | rs1288480 | 1 | 53549204 | 0.38 | 7804 | intron |  |  |
|  | rs1288495 | 1 | 53535962 | 0.30 | 7804 | intron |  |  |
|  | rs1288502 | 1 | 53529742 | 0.48 | 7804 | intron |  |  |
|  | rs1288504 | 1 | 53529330 | 0.33 | 7804 | intron |  |  |
|  | rs1288516 | 1 | 53520874 | 0.39 | 7804 | intron |  |  |
|  | rs1288519 | 1 | 53558737 | 0.36 | 7804 | intron |  |  |
|  | rs17108202 | 1 | 53527479 | 0.38 | 7804 | intron |  |  |
|  | rs2782500 | 1 | 53537223 | 0.21 | 7804 | intron |  |  |
|  | rs5177 | 1 | 53484323 | 0.28 | 7804 | 3UTR |  |  |
| Melanocortin 3 receptor | rs3746619 | 20 | 54257212 | 0.12 | 4159 | coding |  |  |
|  | rs3827103 | 20 | 54257436 | 0.10 | 4159 | coding |  |  |
|  | rs6092276 | 20 | 54255495 | 0.31 | 4159 | flanking_5UTR |  |  |
|  | rs6127698 | 20 | 54256823 | 0.42 | 4159 | flanking_5UTR |  |  |
| Melanocortin 4 receptor | rs8087522 | 18 | 56191458 | 0.28 | 4160 | flanking_5UTR |  |  |
| Melanin-concentrating hormone receptor 2 | rs10499026 | 6 | 100504247 | 0.22 | 84539 | intron |  |  |
|  | rs13206575 | 6 | 100476005 | 0.26 | 84539 | intron |  |  |
|  | rs2001456 | 6 | 100545730 | 0.22 | 728012 | intron |  |  |
|  | rs2397435 | 6 | 100494977 | 0.34 | 84539 | intron |  |  |
|  | rs4448104 | 6 | 100489440 | 0.22 | 84539 | intron |  |  |
|  | rs4559096 | 6 | 100498593 | 0.35 | 84539 | intron |  |  |
|  | rs4565313 | 6 | 100489599 | 0.22 | 84539 | intron |  |  |
|  | rs4840106 | 6 | 100510235 | 0.33 | 84539 | intron |  |  |
|  | rs7760129 | 6 | 100530325 | 0.26 | 728012 | flanking_5UTR |  |  |
|  | rs9373294 | 6 | 100488421 | 0.34 | 84539 | intron |  |  |
|  | rs9376618 | 6 | 100522273 | 0.24 | 728012 | flanking_5UTR |  |  |
|  | rs9389906 | 6 | 100513917 | 0.40 | 84539 | flanking_5UTR |  |  |
|  | rs9496085 | 6 | 100537524 | 0.39 | 728012 | intron |  |  |
| Multiple EGF-like-domains 8 | rs2304210 | 19 | 47574693 | 0.11 | 90198 | coding |  |  |
| Melanoma inhibitory activity family, member 3 | rs17163313 | 1 | 220866248 | 0.30 | 375056 | intron |  |  |
|  | rs17465637 | 1 | 220890152 | 0.30 | 375056 | intron |  |  |
|  | rs2378584 | 1 | 220860255 | 0.30 | 375056 | intron |  |  |
|  | rs2936051 | 1 | 220869827 | 0.20 | 375056 | coding |  |  |
|  | rs2936052 | 1 | 220868999 | 0.20 | 375056 | coding |  |  |
| Matrix metallopeptidase 3 | rs520540 | 11 | 102214635 | 0.40 | 4314 | coding |  |  |
|  | rs569444 | 11 | 102212515 | 0.14 | 4314 | intron |  |  |
|  | rs650108 | 11 | 102213997 | 0.33 | 4314 | intron |  |  |
| Matrix metallopeptidase 9 | rs2274756 | 20 | 44076518 | 0.16 | 4318 | coding |  |  |
| Methylenetetrahydrofolate dehydrogenase | rs1047665 | 6 | 151464648 | 0.11 | 25902 | 3UTR |  |  |
|  | rs11155760 | 6 | 151296407 | 0.35 | 644860 | flanking_5UTR |  |  |
|  | rs11155763 | 6 | 151324570 | 0.45 | 25902 | intron |  |  |
|  | rs11753794 | 6 | 151460013 | 0.34 | 646104 | intron |  |  |
|  | rs12211869 | 6 | 151430822 | 0.25 | 25902 | intron |  |  |
|  | rs12524884 | 6 | 151435807 | 0.12 | 25902 | intron |  |  |
|  | rs13199827 | 6 | 151461499 | 0.26 | 646104 | intron |  |  |
|  | rs13214952 | 6 | 151401751 | 0.32 | 25902 | intron |  |  |
|  | rs1555179 | 6 | 151313049 | 0.29 | 25902 | intron |  |  |
|  | rs1555182 | 6 | 151338224 | 0.43 | 25902 | intron |  |  |
|  | rs1570190 | 6 | 151428908 | 0.09 | 25902 | intron |  |  |
|  | rs1575219 | 6 | 151437721 | 0.15 | 25902 | intron |  |  |
|  | rs17080476 | 6 | 151271690 | 0.14 | 25902 | intron |  |  |
|  | rs17080757 | 6 | 151449165 | 0.30 | 25902 | intron |  |  |
|  | rs17349743 | 6 | 151254904 | 0.25 | 25902 | intron |  |  |
|  | rs1738583 | 6 | 151320719 | 0.12 | 25902 | intron |  |  |
|  | rs175853 | 6 | 151287007 | 0.37 | 25902 | intron |  |  |
|  | rs175862 | 6 | 151273763 | 0.22 | 25902 | intron |  |  |
|  | rs175866 | 6 | 151267214 | 0.45 | 25902 | intron |  |  |
|  | rs1771798 | 6 | 151323260 | 0.08 | 25902 | intron |  |  |
|  | rs1771845 | 6 | 151272530 | 0.27 | 25902 | intron |  |  |
|  | rs2073066 | 6 | 151239424 | 0.20 | 25902 | intron |  |  |
|  | rs2073067 | 6 | 151238845 | 0.34 | 25902 | intron |  |  |
|  | rs2096066 | 6 | 151297127 | 0.48 | 644860 | flanking_5UTR |  |  |
|  | rs2105286 | 6 | 151297245 | 0.18 | 644860 | flanking_5UTR |  |  |
|  | rs2295084 | 6 | 151284927 | 0.22 | 25902 | intron |  |  |
|  | rs2839947 | 6 | 151230384 | 0.40 | 25902 | intron |  |  |
|  | rs3818056 | 6 | 151377388 | 0.40 | 25902 | intron |  |  |
|  | rs4341013 | 6 | 151450554 | 0.20 | 25902 | intron |  |  |
|  | rs472703 | 6 | 151382991 | 0.17 | 25902 | intron |  |  |
|  | rs4869708 | 6 | 151341448 | 0.45 | 25902 | intron |  |  |
|  | rs4869713 | 6 | 151430560 | 0.33 | 25902 | intron |  |  |
|  | rs4869959 | 6 | 151303232 | 0.20 | 25902 | intron |  |  |
|  | rs487637 | 6 | 151381135 | 0.24 | 25902 | intron |  |  |
|  | rs497161 | 6 | 151363868 | 0.45 | 25902 | intron |  |  |
|  | rs505358 | 6 | 151369541 | 0.36 | 25902 | intron |  |  |
|  | rs507108 | 6 | 151369385 | 0.17 | 25902 | intron |  |  |
|  | rs510957 | 6 | 151383747 | 0.46 | 25902 | intron |  |  |
|  | rs524732 | 6 | 151356534 | 0.24 | 25902 | intron |  |  |
|  | rs529087 | 6 | 151369252 | 0.29 | 25902 | intron |  |  |
|  | rs538017 | 6 | 151358885 | 0.26 | 25902 | intron |  |  |
|  | rs558936 | 6 | 151331135 | 0.32 | 25902 | intron |  |  |
|  | rs563440 | 6 | 151330684 | 0.21 | 25902 | intron |  |  |
|  | rs572522 | 6 | 151348014 | 0.44 | 25902 | intron |  |  |
|  | rs6905272 | 6 | 151269402 | 0.11 | 25902 | intron |  |  |
|  | rs6912618 | 6 | 151436081 | 0.14 | 25902 | intron |  |  |
|  | rs6913898 | 6 | 151423570 | 0.41 | 25902 | intron |  |  |
|  | rs6922248 | 6 | 151294655 | 0.29 | 644860 | flanking_5UTR |  |  |
|  | rs6936332 | 6 | 151341100 | 0.43 | 25902 | intron |  |  |
|  | rs6940322 | 6 | 151236228 | 0.35 | 25902 | intron |  |  |
|  | rs712208 | 6 | 151269980 | 0.17 | 25902 | intron |  |  |
|  | rs742829 | 6 | 151300463 | 0.14 | 25902 | intron |  |  |
|  | rs7739248 | 6 | 151323361 | 0.27 | 25902 | intron |  |  |
|  | rs7763414 | 6 | 151447070 | 0.17 | 25902 | intron |  |  |
|  | rs7765521 | 6 | 151246236 | 0.44 | 25902 | intron |  |  |
|  | rs803422 | 6 | 151257287 | 0.28 | 25902 | intron |  |  |
|  | rs803446 | 6 | 151306907 | 0.30 | 25902 | intron |  |  |
|  | rs803450 | 6 | 151305227 | 0.39 | 25902 | intron |  |  |
|  | rs803456 | 6 | 151287681 | 0.37 | 25902 | intron |  |  |
|  | rs803470 | 6 | 151312188 | 0.12 | 25902 | intron |  |  |
|  | rs803471 | 6 | 151312062 | 0.30 | 25902 | intron |  |  |
|  | rs9322300 | 6 | 151416348 | 0.49 | 25902 | intron |  |  |
|  | rs9322301 | 6 | 151433014 | 0.42 | 25902 | intron |  |  |
|  | rs9371491 | 6 | 151415348 | 0.12 | 25902 | intron |  |  |
|  | rs9371494 | 6 | 151444841 | 0.31 | 25902 | intron |  |  |
|  | rs9397032 | 6 | 151438384 | 0.35 | 25902 | intron |  |  |
|  | rs9397033 | 6 | 151456785 | 0.43 | 25902 | flanking_3UTR |  |  |
|  | rs9397365 | 6 | 151277873 | 0.11 | 25902 | intron |  |  |
|  | rs9478878 | 6 | 151334970 | 0.31 | 25902 | intron |  |  |
|  | rs9478908 | 6 | 151364141 | 0.32 | 25902 | intron |  |  |
|  | rs9968875 | 6 | 151355060 | 0.10 | 25902 | intron |  |  |
|  | rs997429 | 6 | 151265373 | 0.22 | 25902 | intron |  |  |
| Methylenetetrahydrofolate reductase | rs12121543 | 1 | 11777258 | 0.25 | 4524 | intron |  |  |
|  | rs1476413 | 1 | 11774887 | 0.28 | 4524 | intron |  |  |
|  | rs17367504 | 1 | 11785365 | 0.12 | 4524 | intron |  |  |
|  | rs1801131 | 1 | 11777063 | 0.34 | 4524 | coding |  |  |
|  | rs1801133 | 1 | 11778965 | 0.37 | 4524 | coding |  |  |
|  | rs2066471 | 1 | 11783045 | 0.23 | 4524 | intron |  |  |
|  | rs4846048 | 1 | 11768839 | 0.39 | 4524 | 3UTR |  |  |
|  | rs6541003 | 1 | 11778454 | 0.47 | 4524 | intron |  |  |
|  | rs9651118 | 1 | 11784801 | 0.09 | 4524 | intron |  |  |
| Nitric oxide synthase 3 | rs1799983 | 7 | 150327044 | 0.23 | 4846 | coding |  |  |
|  | rs1800783 | 7 | 150320330 | 0.40 | 4846 | intron |  |  |
|  | rs3918188 | 7 | 150333714 | 0.40 | 4846 | intron |  |  |
|  | rs3918227 | 7 | 150331879 | 0.03 | 4846 | intron |  |  |
|  | rs743507 | 7 | 150338421 | 0.27 | 4846 | intron |  |  |
| Neuropeptide Y | rs16129 | 7 | 24297290 | 0.48 | 4852 | intron |  |  |
|  | rs16131 | 7 | 24296362 | 0.13 | 4852 | intron |  |  |
|  | rs16138 | 7 | 24292020 | 0.29 | 4852 | intron |  |  |
| Neuropeptide Y receptor Y1 | rs9764 | 4 | 164464855 | 0.25 | 4886 | 3UTR |  |  |
|  | rs4691075 | 4 | 164468935 | 0.16 | 4886 | flanking_5UTR |  |  |
|  | rs7687423 | 4 | 164470247 | 0.49 | 729743 | flanking_3UTR |  |  |
| Neuropeptide Y receptor Y2 | rs10461238 | 4 | 156351666 | 0.39 | 4887 | intron |  |  |
|  | rs10461239 | 4 | 156351897 | 0.08 | 4887 | intron |  |  |
|  | rs1047214 | 4 | 156355126 | 0.49 | 4887 | coding |  |  |
|  | rs12507396 | 4 | 156348494 | 0.08 | 4887 | flanking_5UTR |  |  |
|  | rs17304901 | 4 | 156357822 | 0.30 | 4887 | flanking_3UTR |  |  |
|  | rs2234759 | 4 | 156349007 | 0.16 | 4887 | flanking_5UTR |  |  |
|  | rs6857715 | 4 | 156348632 | 0.45 | 4887 | flanking_5UTR |  |  |
| Neuropeptide Y receptor Y5 | rs11100494 | 4 | 164489703 | 0.06 | 4889 | intron |  |  |
|  | rs11724320 | 4 | 164487373 | 0.45 | 4889 | intron |  |  |
|  | rs4632602 | 4 | 164486579 | 0.17 | 4889 | intron |  |  |
|  | rs7679206 | 4 | 164488684 | 0.31 | 4889 | intron |  |  |
| Neuropeptide Y receptor Y6 | rs12652106 | 5 | 137170752 | 0.07 | 4888 | flanking_5UTR |  |  |
| Nuclear receptor subfamily 0, group B, member 2 | rs17162330 | 1 | 27108799 | 0.09 | 8431 | flanking_3UTR |  |  |
|  | rs6659176 | 1 | 27112507 | 0.04 | 8431 | coding |  |  |
|  | rs7504 | 1 | 27110737 | 0.26 | 8431 | 3UTR |  |  |
| Glucocorticoid receptor | rs10041520 | 5 | 142756877 | 0.49 | 2908 | flanking_3UTR |  |  |
|  | rs10482616 | 5 | 142761760 | 0.20 | 2908 | intron |  |  |
|  | rs10482682 | 5 | 142659590 | 0.47 | 2908 | intron |  |  |
|  | rs17209251 | 5 | 142649416 | 0.16 | 2908 | intron |  |  |
|  | rs2918417 | 5 | 142706363 | 0.25 | 729123 | flanking_3UTR |  |  |
|  | rs2963151 | 5 | 142714344 | 0.14 | 729123 | flanking_3UTR |  |  |
|  | rs2963155 | 5 | 142736197 | 0.16 | 2908 | flanking_3UTR |  |  |
|  | rs4128428 | 5 | 142761813 | 0.11 | 2908 | intron |  |  |
|  | rs4912905 | 5 | 142710569 | 0.23 | 729123 | flanking_3UTR |  |  |
| Neurotrophic tyrosine kinase receptor type 1 | rs10908521 | 1 | 155080274 | 0.29 | 3645 | intron |  |  |
|  | rs12132885 | 1 | 155108960 | 0.34 | 4914 | intron |  |  |
|  | rs1800601 | 1 | 155052241 | 0.32 | 4914 | 5UTR |  |  |
|  | rs1800879 | 1 | 155104765 | 0.29 | 4914 | intron |  |  |
|  | rs1998977 | 1 | 155070745 | 0.38 | 3645 | flanking_3UTR |  |  |
|  | rs2768755 | 1 | 155114744 | 0.29 | 4914 | intron |  |  |
|  | rs4661061 | 1 | 155069281 | 0.21 | 3645 | flanking_3UTR |  |  |
|  | rs4661063 | 1 | 155091425 | 0.17 | 3645 | intron |  |  |
|  | rs4661229 | 1 | 155075830 | 0.17 | 3645 | flanking_3UTR |  |  |
|  | rs6334 | 1 | 155112857 | 0.20 | 4914 | coding |  |  |
| One cut homeobox 1 | rs12101908 | 15 | 50851526 | 0.35 | 3175 | intron |  |  |
|  | rs17543338 | 15 | 50857166 | 0.10 | 3175 | intron |  |  |
|  | rs1899744 | 15 | 50845703 | 0.45 | 3175 | intron |  |  |
|  | rs2165990 | 15 | 50857039 | 0.33 | 3175 | intron |  |  |
|  | rs2440325 | 15 | 50853090 | 0.47 | 3175 | intron |  |  |
|  | rs2440326 | 15 | 50849028 | 0.34 | 3175 | intron |  |  |
|  | rs7180600 | 15 | 50857433 | 0.12 | 3175 | intron |  |  |
| Poly(A) binding protein interacting protein 1 | rs10462051 | 5 | 43581067 | 0.40 | 10605 | intron |  |  |
|  | rs6866325 | 5 | 43565792 | 0.47 | 10605 | intron |  |  |
| Pregnancy-associated plasma protein A | rs10120448 | 9 | 118150261 | 0.24 | 5069 | intron |  |  |
|  | rs10435873 | 9 | 118077855 | 0.47 | 5069 | intron |  |  |
|  | rs10513272 | 9 | 118103868 | 0.10 | 5069 | intron |  |  |
|  | rs1054402 | 9 | 118203330 | 0.31 | 5069 | 3UTR |  |  |
|  | rs10817865 | 9 | 118018503 | 0.50 | 5069 | intron |  |  |
|  | rs10817881 | 9 | 118180309 | 0.30 | 5069 | intron |  |  |
|  | rs10983088 | 9 | 118051212 | 0.28 | 5069 | intron |  |  |
|  | rs10983127 | 9 | 118185021 | 0.14 | 5069 | intron |  |  |
|  | rs10983132 | 9 | 118189261 | 0.24 | 5069 | intron |  |  |
|  | rs11790784 | 9 | 117965932 | 0.04 | 5069 | intron |  |  |
|  | rs12379469 | 9 | 118010252 | 0.24 | 5069 | intron |  |  |
|  | rs13298102 | 9 | 118062623 | 0.26 | 5069 | intron |  |  |
|  | rs1331140 | 9 | 118067539 | 0.41 | 5069 | intron |  |  |
|  | rs1331141 | 9 | 118066405 | 0.16 | 5069 | intron |  |  |
|  | rs1354956 | 9 | 118193373 | 0.42 | 5069 | intron |  |  |
|  | rs1411820 | 9 | 118066728 | 0.46 | 5069 | intron |  |  |
|  | rs1411821 | 9 | 117974868 | 0.19 | 5069 | intron |  |  |
|  | rs1570518 | 9 | 118118897 | 0.25 | 5069 | intron |  |  |
|  | rs1998499 | 9 | 118115763 | 0.12 | 5069 | intron |  |  |
|  | rs2418444 | 9 | 118145488 | 0.13 | 5069 | intron |  |  |
|  | rs374560 | 9 | 118020365 | 0.39 | 5069 | intron |  |  |
|  | rs3761843 | 9 | 118136010 | 0.27 | 5069 | intron |  |  |
|  | rs3789280 | 9 | 117993193 | 0.27 | 5069 | intron |  |  |
|  | rs3789281 | 9 | 117993278 | 0.47 | 5069 | intron |  |  |
|  | rs3789299 | 9 | 118166791 | 0.48 | 5069 | intron |  |  |
|  | rs3810939 | 9 | 118200612 | 0.28 | 5069 | 3UTR |  |  |
|  | rs386088 | 9 | 117956014 | 0.43 | 5069 | 5UTR |  |  |
|  | rs390623 | 9 | 118028734 | 0.15 | 5069 | intron |  |  |
|  | rs4837294 | 9 | 118074296 | 0.13 | 5069 | intron |  |  |
|  | rs4837374 | 9 | 118194437 | 0.22 | 5069 | intron |  |  |
|  | rs4837525 | 9 | 118078379 | 0.35 | 5069 | intron |  |  |
|  | rs7026755 | 9 | 118195909 | 0.45 | 5069 | intron |  |  |
|  | rs7390972 | 9 | 118168396 | 0.37 | 5069 | intron |  |  |
|  | rs751543 | 9 | 118162163 | 0.22 | 5069 | intron |  |  |
|  | rs756624 | 9 | 118032202 | 0.48 | 5069 | intron |  |  |
|  | rs7853634 | 9 | 118161874 | 0.10 | 5069 | intron |  |  |
|  | rs7874262 | 9 | 118080839 | 0.07 | 5069 | intron |  |  |
|  | rs912214 | 9 | 118147508 | 0.25 | 5069 | intron |  |  |
|  | rs932705 | 9 | 118187575 | 0.23 | 5069 | intron |  |  |
|  | rs9695235 | 9 | 118177949 | 0.16 | 5069 | intron |  |  |
|  | rs978201 | 9 | 118196851 | 0.36 | 5069 | intron |  |  |
| Period homolog 3 | rs10462018 | 1 | 7802214 | 0.04 | 8863 | intron |  |  |
|  | rs1891217 | 1 | 7794436 | 0.16 | 8863 | intron |  |  |
|  | rs2172563 | 1 | 7796630 | 0.23 | 8863 | intron |  |  |
|  | rs228665 | 1 | 7813543 | 0.42 | 8863 | intron |  |  |
|  | rs228682 | 1 | 7778933 | 0.44 | 8863 | intron |  |  |
|  | rs228691 | 1 | 7803056 | 0.29 | 8863 | intron |  |  |
|  | rs2640909 | 1 | 7812704 | 0.28 | 8863 | coding |  |  |
|  | rs697686 | 1 | 7772805 | 0.29 | 8863 | intron |  |  |
|  | rs697693 | 1 | 7809011 | 0.23 | 8863 | intron |  |  |
|  | rs707472 | 1 | 7828595 | 0.23 | 8863 | flanking_3UTR |  |  |
|  | rs875994 | 1 | 7775506 | 0.09 | 8863 | intron |  |  |
| Perilipin 1 | rs1052700 | 15 | 88009314 | 0.41 | 5346 | 3UTR |  |  |
|  | rs894160 | 15 | 88012827 | 0.39 | 5346 | intron |  |  |
| Phenylethanolamine N-methyltransferase | rs1107794 | 17 | 35077126 | 0.08 | 728108 | flanking_3UTR |  |  |
| Proopionelanocortin | rs6713532 | 2 | 25238337 | 0.25 | 5443 | intron |  |  |
|  | rs7565427 | 2 | 25239142 | 0.16 | 5443 | intron |  |  |
|  | rs7565877 | 2 | 25239568 | 0.12 | 5443 | intron |  |  |
|  | rs934778 | 2 | 25242728 | 0.28 | 5443 | flanking_5UTR |  |  |
| Paraoxonase 2 | rs11981433 | 7 | 94892276 | 0.47 | 5445 | intron |  |  |
|  | rs2237585 | 7 | 94887754 | 0.39 | 5445 | intron |  |  |
|  | rs2299266 | 7 | 94887009 | 0.20 | 5445 | intron |  |  |
|  | rs2299267 | 7 | 94899857 | 0.17 | 5445 | intron |  |  |
|  | rs730365 | 7 | 94900936 | 0.18 | 5445 | intron |  |  |
|  | rs7802018 | 7 | 94898249 | 0.37 | 5445 | intron |  |  |
|  | rs9641164 | 7 | 94872757 | 0.17 | 5445 | intron |  |  |
| Peroxisome proliferator-activated receptor α | rs11703495 | 22 | 44972000 | 0.10 | 5465 | intron |  |  |
|  | rs12330015 | 22 | 44968942 | 0.13 | 5465 | intron |  |  |
|  | rs129600 | 22 | 44935825 | 0.21 | 5465 | intron |  |  |
|  | rs135547 | 22 | 44932314 | 0.22 | 5465 | intron |  |  |
|  | rs135551 | 22 | 44931685 | 0.18 | 5465 | intron |  |  |
|  | rs4253623 | 22 | 44928770 | 0.09 | 5465 | intron |  |  |
|  | rs4253655 | 22 | 44947835 | 0.20 | 5465 | intron |  |  |
|  | rs4253701 | 22 | 44964774 | 0.18 | 5465 | intron |  |  |
|  | rs4253712 | 22 | 44973699 | 0.22 | 5465 | intron |  |  |
|  | rs4253747 | 22 | 44991901 | 0.20 | 5465 | intron |  |  |
|  | rs4253760 | 22 | 45001048 | 0.18 | 5465 | intron |  |  |
|  | rs4253776 | 22 | 45008143 | 0.13 | 5465 | intron |  |  |
|  | rs5766743 | 22 | 44986042 | 0.31 | 5465 | intron |  |  |
|  | rs6007662 | 22 | 44999709 | 0.23 | 5465 | intron |  |  |
|  | rs6008197 | 22 | 44999080 | 0.14 | 5465 | intron |  |  |
|  | rs6008259 | 22 | 45012446 | 0.17 | 5465 | 3UTR |  |  |
|  | rs8138102 | 22 | 44970416 | 0.26 | 5465 | intron |  |  |
|  | rs9626736 | 22 | 44948896 | 0.38 | 5465 | intron |  |  |
| Peroxisome proliferator-activated receptor δ | rs2038068 | 6 | 35482439 | 0.40 | 5467 | intron |  |  |
|  | rs2267668 | 6 | 35485900 | 0.21 | 5467 | intron |  |  |
|  | rs9658119 | 6 | 35473945 | 0.29 | 5467 | intron |  |  |
| Peroxisome proliferator-activated receptor γ | rs1151996 | 3 | 12420807 | 0.39 | 5468 | intron |  |  |
|  | rs1175540 | 3 | 12440243 | 0.37 | 5468 | intron |  |  |
|  | rs13076055 | 3 | 12316996 | 0.22 | 5468 | intron |  |  |
|  | rs13099828 | 3 | 12443589 | 0.21 | 5468 | intron |  |  |
|  | rs17793693 | 3 | 12320971 | 0.12 | 5468 | intron |  |  |
|  | rs17793951 | 3 | 12345737 | 0.37 | 5468 | intron |  |  |
|  | rs2938392 | 3 | 12409608 | 0.41 | 5468 | intron |  |  |
|  | rs2972164 | 3 | 12309416 | 0.43 | 5468 | intron |  |  |
|  | rs3856806 | 3 | 12450557 | 0.07 | 5468 | coding |  |  |
|  | rs4135247 | 3 | 12371588 | 0.41 | 5468 | intron |  |  |
|  | rs4135275 | 3 | 12418844 | 0.19 | 5468 | intron |  |  |
|  | rs4135284 | 3 | 12424866 | 0.18 | 5468 | intron |  |  |
|  | rs7626560 | 3 | 12450088 | 0.20 | 5468 | intron |  |  |
| Peroxisome proliferator-activated receptor γ, coactivator 1α | rs10002477 | 4 | 23466142 | 0.39 | 10891 | intron |  |  |
|  | rs10213440 | 4 | 23475437 | 0.20 | 10891 | intron |  |  |
|  | rs11724368 | 4 | 23472031 | 0.28 | 10891 | intron |  |  |
|  | rs11734408 | 4 | 23491617 | 0.18 | 10891 | intron |  |  |
|  | rs11941854 | 4 | 23468058 | 0.07 | 10891 | intron |  |  |
|  | rs12374408 | 4 | 23463581 | 0.35 | 10891 | intron |  |  |
|  | rs12500214 | 4 | 23494666 | 0.27 | 10891 | intron |  |  |
|  | rs1472095 | 4 | 23427175 | 0.18 | 10891 | intron |  |  |
|  | rs2932965 | 4 | 23414584 | 0.18 | 10891 | intron |  |  |
|  | rs2946385 | 4 | 23495421 | 0.50 | 10891 | intron |  |  |
|  | rs2970849 | 4 | 23426905 | 0.26 | 10891 | intron |  |  |
|  | rs2970853 | 4 | 23432617 | 0.23 | 10891 | intron |  |  |
|  | rs2970872 | 4 | 23498911 | 0.44 | 10891 | intron |  |  |
|  | rs3736265 | 4 | 23423805 | 0.08 | 10891 | coding |  |  |
|  | rs3755863 | 4 | 23424620 | 0.47 | 10891 | coding |  |  |
|  | rs3774908 | 4 | 23438032 | 0.08 | 10891 | intron |  |  |
|  | rs3774921 | 4 | 23419745 | 0.35 | 10891 | intron |  |  |
|  | rs3796407 | 4 | 23493809 | 0.30 | 10891 | intron |  |  |
|  | rs4235308 | 4 | 23473510 | 0.48 | 10891 | intron |  |  |
|  | rs4361373 | 4 | 23484773 | 0.18 | 10891 | intron |  |  |
|  | rs4550905 | 4 | 23484657 | 0.37 | 10891 | intron |  |  |
|  | rs4697046 | 4 | 23445498 | 0.34 | 10891 | intron |  |  |
|  | rs6850464 | 4 | 23468580 | 0.12 | 10891 | intron |  |  |
|  | rs7437482 | 4 | 23465072 | 0.28 | 10891 | intron |  |  |
|  | rs7656250 | 4 | 23475114 | 0.31 | 10891 | intron |  |  |
|  | rs7665116 | 4 | 23462109 | 0.18 | 10891 | intron |  |  |
|  | rs8192678 | 4 | 23424760 | 0.35 | 10891 | coding |  |  |
| Peroxisome proliferator-activated receptor γ, coactivator 1β | rs10036538 | 5 | 149135781 | 0.29 | 133522 | intron |  |  |
|  | rs10080122 | 5 | 149099034 | 0.21 | 133522 | intron |  |  |
|  | rs1012543 | 5 | 149157138 | 0.28 | 133522 | intron |  |  |
|  | rs1038071 | 5 | 149103348 | 0.11 | 133522 | intron |  |  |
|  | rs10875551 | 5 | 149153327 | 0.20 | 133522 | intron |  |  |
|  | rs10875552 | 5 | 149169682 | 0.35 | 133522 | intron |  |  |
|  | rs13167468 | 5 | 149133236 | 0.14 | 133522 | intron |  |  |
|  | rs13174179 | 5 | 149130864 | 0.35 | 133522 | intron |  |  |
|  | rs1422429 | 5 | 149146627 | 0.45 | 133522 | intron |  |  |
|  | rs1544744 | 5 | 149167231 | 0.09 | 133522 | intron |  |  |
|  | rs17461842 | 5 | 149159918 | 0.13 | 133522 | intron |  |  |
|  | rs17462080 | 5 | 149180595 | 0.12 | 133522 | intron |  |  |
|  | rs17653473 | 5 | 149163569 | 0.30 | 133522 | intron |  |  |
|  | rs17654872 | 5 | 149095344 | 0.16 | 133522 | intron |  |  |
|  | rs2010994 | 5 | 149171845 | 0.24 | 133522 | intron |  |  |
|  | rs2161257 | 5 | 149170190 | 0.47 | 133522 | intron |  |  |
|  | rs2340621 | 5 | 149122509 | 0.32 | 133522 | intron |  |  |
|  | rs251460 | 5 | 149177423 | 0.28 | 133522 | intron |  |  |
|  | rs32587 | 5 | 149180796 | 0.38 | 133522 | intron |  |  |
|  | rs4705090 | 5 | 149125062 | 0.23 | 133522 | intron |  |  |
|  | rs4705374 | 5 | 149131512 | 0.16 | 133522 | intron |  |  |
|  | rs4705378 | 5 | 149144856 | 0.27 | 133522 | intron |  |  |
|  | rs4705382 | 5 | 149161559 | 0.42 | 133522 | intron |  |  |
|  | rs6579757 | 5 | 149141630 | 0.49 | 133522 | intron |  |  |
|  | rs6888451 | 5 | 149104127 | 0.10 | 133522 | intron |  |  |
|  | rs880770 | 5 | 149135028 | 0.36 | 133522 | intron |  |  |
|  | rs9986116 | 5 | 149093289 | 0.24 | 133522 | intron |  |  |
| Serine protease 57 | rs12609586 | 19 | 640004 | 0.28 | 400668 | intron |  |  |
|  | rs12983321 | 19 | 637344 | 0.10 | 400668 | intron |  |  |
|  | rs4375794 | 19 | 642909 | 0.26 | 400668 | coding |  |  |
| Pregnancy-specific β1-glycoprotein 9 | rs10423013 | 19 | 48450423 | 0.29 | 5678 | intron |  |  |
| Proline/serine-rich coiled-coil 1 | rs14000 | 1 | 109624032 | 0.13 | 84722 | 3UTR |  |  |
|  | rs583104 | 1 | 109622830 | 0.17 | 84722 | flanking_3UTR |  |  |
|  | rs657420 | 1 | 109627659 | 0.45 | 84722 | flanking_5UTR |  |  |
| Peptide YY | rs1058046 | 17 | 39386057 | 0.35 | 5697 | coding |  |  |
|  | rs1618809 | 17 | 39421451 | 0.30 | 5697 | intron |  |  |
|  | rs1642599 | 17 | 39400664 | 0.21 | 5697 | intron |  |  |
|  | rs1859223 | 17 | 39435091 | 0.17 | 5697 | intron |  |  |
| Retinol binding protein 4 | rs13376835 | 10 | 95342163 | 0.27 | 5950 | intron |  |  |
|  | rs7079946 | 10 | 95342575 | 0.31 | 5950 | intron |  |  |
|  | rs7094671 | 10 | 95345607 | 0.37 | 5950 | intron |  |  |
| v-rel reticuloendotheliosis viral oncogene | rs11227247 | 11 | 65179429 | 0.11 | 5970 | intron |  |  |
|  | rs11820062 | 11 | 65186512 | 0.46 | 5970 | intron |  |  |
| Renin | rs10900555 | 1 | 202398933 | 0.41 | 5972 | intron |  |  |
|  | rs11571078 | 1 | 202399797 | 0.16 | 5972 | intron |  |  |
|  | rs1464816 | 1 | 202395477 | 0.32 | 5972 | intron |  |  |
|  | rs2887284 | 1 | 202391271 | 0.29 | 5972 | intron |  |  |
|  | rs5705 | 1 | 202397809 | 0.18 | 5972 | coding |  |  |
|  | rs6693954 | 1 | 202399261 | 0.34 | 5972 | intron |  |  |
| ret proto-oncogene | rs1800860 | 10 | 42926693 | 0.26 | 5979 | coding |  |  |
|  | rs1800863 | 10 | 42935639 | 0.23 | 5979 | coding |  |  |
|  | rs1864402 | 10 | 42925866 | 0.25 | 5979 | intron |  |  |
|  | rs2435342 | 10 | 42904262 | 0.48 | 5979 | intron |  |  |
|  | rs2505515 | 10 | 42925592 | 0.27 | 5979 | intron |  |  |
|  | rs2506020 | 10 | 42899079 | 0.37 | 5979 | intron |  |  |
|  | rs2742234 | 10 | 42932615 | 0.29 | 5979 | intron |  |  |
|  | rs2742236 | 10 | 42940557 | 0.48 | 5979 | intron |  |  |
|  | rs2742240 | 10 | 42944323 | 0.21 | 5979 | 3UTR |  |  |
|  | rs3026737 | 10 | 42916734 | 0.27 | 5979 | intron |  |  |
|  | rs7393733 | 10 | 42908580 | 0.33 | 5979 | intron |  |  |
| Resistin | rs1862513 | 19 | 7639793 | 0.35 | 56729 | flanking_5UTR |  |  |
|  | rs3745367 | 19 | 7640511 | 0.33 | 56729 | intron |  |  |
|  | rs3745369 | 19 | 7641475 | 0.49 | 56729 | flanking_3UTR |  |  |
| SH2 domain containing 2A | rs909200 | 1 | 155044735 | 0.20 | 9047 | intron |  |  |
|  | rs926103 | 1 | 155051606 | 0.39 | 9047 | coding |  |  |
|  | rs928391 | 1 | 155043341 | 0.24 | 9047 | intron |  |  |
| Single-minded homolog 1 | rs1395119 | 6 | 100944610 | 0.31 | 6492 | 3UTR |  |  |
|  | rs1847912 | 6 | 100987417 | 0.22 | 6492 | intron |  |  |
|  | rs241809 | 6 | 101010909 | 0.25 | 6492 | intron |  |  |
|  | rs241815 | 6 | 100985179 | 0.23 | 6492 | intron |  |  |
|  | rs241816 | 6 | 100986372 | 0.41 | 6492 | intron |  |  |
|  | rs3734355 | 6 | 100975442 | 0.12 | 6492 | coding |  |  |
|  | rs3778033 | 6 | 100982237 | 0.25 | 6492 | intron |  |  |
|  | rs3798490 | 6 | 101000900 | 0.23 | 6492 | intron |  |  |
|  | rs3798497 | 6 | 100985579 | 0.18 | 6492 | intron |  |  |
|  | rs3798500 | 6 | 100969148 | 0.05 | 6492 | intron |  |  |
|  | rs3798508 | 6 | 100960685 | 0.20 | 6492 | intron |  |  |
|  | rs3798514 | 6 | 100950989 | 0.13 | 6492 | intron |  |  |
|  | rs714378 | 6 | 100979508 | 0.18 | 6492 | intron |  |  |
|  | rs9376963 | 6 | 100955040 | 0.26 | 6492 | intron |  |  |
| Solute carrier family 2 | rs12407920 | 1 | 43190368 | 0.10 | 6513 | intron |  |  |
|  | rs3754219 | 1 | 43172273 | 0.42 | 6513 | intron |  |  |
|  | rs3768029 | 1 | 43171574 | 0.43 | 6513 | intron |  |  |
|  | rs3820546 | 1 | 43170672 | 0.47 | 6513 | intron |  |  |
|  | rs3820548 | 1 | 43170983 | 0.30 | 6513 | intron |  |  |
|  | rs710221 | 1 | 43186787 | 0.44 | 6513 | intron |  |  |
|  | rs751210 | 1 | 43183446 | 0.37 | 6513 | intron |  |  |
|  | rs841853 | 1 | 43174025 | 0.32 | 6513 | intron |  |  |
|  | rs841856 | 1 | 43172700 | 0.19 | 6513 | intron |  |  |
|  | rs841858 | 1 | 43171754 | 0.16 | 6513 | intron |  |  |
|  | rs16956647 | 17 | 7126746 | 0.04 | 6517 | intron |  |  |
|  | rs5435 | 17 | 7127847 | 0.28 | 6517 | coding |  |  |
| Slit homolog 2 | rs10019416 | 4 | 20096809 | 0.22 | 9353 | intron |  |  |
|  | rs1033111 | 4 | 20076865 | 0.44 | 9353 | intron |  |  |
|  | rs10516357 | 4 | 20172206 | 0.20 | 9353 | intron |  |  |
|  | rs10938796 | 4 | 19936108 | 0.44 | 9353 | intron |  |  |
|  | rs11733205 | 4 | 20067786 | 0.36 | 9353 | intron |  |  |
|  | rs11933090 | 4 | 20055288 | 0.04 | 9353 | intron |  |  |
|  | rs11936122 | 4 | 20082376 | 0.17 | 9353 | intron |  |  |
|  | rs11942897 | 4 | 20068216 | 0.49 | 9353 | intron |  |  |
|  | rs13134750 | 4 | 19969408 | 0.15 | 9353 | intron |  |  |
|  | rs13140329 | 4 | 19917020 | 0.40 | 9353 | intron |  |  |
|  | rs1379659 | 4 | 20229781 | 0.15 | 9353 | 3UTR |  |  |
|  | rs16869595 | 4 | 20046230 | 0.12 | 9353 | intron |  |  |
|  | rs16869643 | 4 | 20088718 | 0.23 | 9353 | intron |  |  |
|  | rs16869706 | 4 | 20133813 | 0.19 | 9353 | intron |  |  |
|  | rs17534458 | 4 | 19870884 | 0.41 | 9353 | intron |  |  |
|  | rs17537997 | 4 | 20042850 | 0.22 | 9353 | intron |  |  |
|  | rs17544103 | 4 | 20228988 | 0.05 | 9353 | intron |  |  |
|  | rs17601458 | 4 | 19892167 | 0.35 | 9353 | intron |  |  |
|  | rs17620615 | 4 | 20179885 | 0.20 | 9353 | intron |  |  |
|  | rs1923839 | 4 | 19871907 | 0.27 | 9353 | intron |  |  |
|  | rs1995982 | 4 | 20033872 | 0.25 | 9353 | intron |  |  |
|  | rs2031141 | 4 | 19937532 | 0.12 | 9353 | intron |  |  |
|  | rs2168802 | 4 | 20194981 | 0.10 | 9353 | intron |  |  |
|  | rs2292439 | 4 | 20156936 | 0.36 | 9353 | intron |  |  |
|  | rs2322559 | 4 | 20214692 | 0.37 | 9353 | intron |  |  |
|  | rs3775815 | 4 | 20138768 | 0.15 | 9353 | intron |  |  |
|  | rs3775816 | 4 | 20144815 | 0.34 | 9353 | intron |  |  |
|  | rs3775823 | 4 | 20151583 | 0.23 | 9353 | intron |  |  |
|  | rs3775824 | 4 | 20154010 | 0.23 | 9353 | intron |  |  |
|  | rs3775827 | 4 | 20154412 | 0.16 | 9353 | intron |  |  |
|  | rs4351015 | 4 | 19960276 | 0.31 | 9353 | intron |  |  |
|  | rs4552454 | 4 | 19995142 | 0.10 | 9353 | intron |  |  |
|  | rs4696953 | 4 | 19932011 | 0.38 | 9353 | intron |  |  |
|  | rs491795 | 4 | 20087382 | 0.16 | 9353 | intron |  |  |
|  | rs5016268 | 4 | 19979241 | 0.37 | 9353 | intron |  |  |
|  | rs520535 | 4 | 20144974 | 0.41 | 9353 | intron |  |  |
|  | rs522501 | 4 | 20106375 | 0.24 | 9353 | intron |  |  |
|  | rs525684 | 4 | 20103359 | 0.48 | 9353 | intron |  |  |
|  | rs556816 | 4 | 20111543 | 0.47 | 9353 | intron |  |  |
|  | rs569230 | 4 | 20098122 | 0.38 | 9353 | intron |  |  |
|  | rs573118 | 4 | 20143784 | 0.13 | 9353 | intron |  |  |
|  | rs575088 | 4 | 20166557 | 0.41 | 9353 | intron |  |  |
|  | rs587032 | 4 | 19880871 | 0.13 | 9353 | intron |  |  |
|  | rs615576 | 4 | 19907507 | 0.11 | 9353 | intron |  |  |
|  | rs636798 | 4 | 20161255 | 0.36 | 9353 | intron |  |  |
|  | rs644607 | 4 | 20180434 | 0.09 | 9353 | intron |  |  |
|  | rs6447952 | 4 | 19885350 | 0.32 | 9353 | intron |  |  |
|  | rs649424 | 4 | 20154179 | 0.28 | 9353 | intron |  |  |
|  | rs6816854 | 4 | 20143565 | 0.46 | 9353 | intron |  |  |
|  | rs6825654 | 4 | 20127701 | 0.37 | 9353 | intron |  |  |
|  | rs6828506 | 4 | 19900021 | 0.25 | 9353 | intron |  |  |
|  | rs729918 | 4 | 20115199 | 0.38 | 9353 | intron |  |  |
|  | rs7668974 | 4 | 19921095 | 0.42 | 9353 | intron |  |  |
|  | rs7669094 | 4 | 20153514 | 0.23 | 9353 | intron |  |  |
|  | rs7680377 | 4 | 19888956 | 0.31 | 9353 | intron |  |  |
|  | rs7690492 | 4 | 20153282 | 0.25 | 9353 | coding |  |  |
|  | rs7690660 | 4 | 20136962 | 0.18 | 9353 | intron |  |  |
|  | rs884770 | 4 | 20056138 | 0.19 | 9353 | intron |  |  |
|  | rs9992559 | 4 | 20139472 | 0.28 | 9353 | intron |  |  |
|  | rs9992591 | 4 | 20202844 | 0.37 | 9353 | intron |  |  |
| SMAD family member 3 | rs11071933 | 15 | 65188800 | 0.34 | 4088 | intron |  |  |
|  | rs11634793 | 15 | 65234506 | 0.62 | 4088 | intron |  |  |
|  | rs11637581 | 15 | 65199103 | 0.19 | 4088 | intron |  |  |
|  | rs11637659 | 15 | 65229440 | 0.21 | 4088 | intron |  |  |
|  | rs11639295 | 15 | 65247811 | 0.38 | 4088 | intron |  |  |
|  | rs12102171 | 15 | 65212087 | 0.25 | 4088 | intron |  |  |
|  | rs12901071 | 15 | 65157443 | 0.33 | 4088 | intron |  |  |
|  | rs12904944 | 15 | 65148828 | 0.33 | 4088 | intron |  |  |
|  | rs12915039 | 15 | 65221402 | 0.27 | 4088 | intron |  |  |
|  | rs1465840 | 15 | 65172545 | 0.39 | 4088 | intron |  |  |
|  | rs1470003 | 15 | 65256389 | 0.42 | 4088 | intron |  |  |
|  | rs1498506 | 15 | 65154688 | 0.47 | 4088 | intron |  |  |
|  | rs1545160 | 15 | 65207772 | 0.36 | 4088 | intron |  |  |
|  | rs17293443 | 15 | 65224917 | 0.20 | 4088 | intron |  |  |
|  | rs1866320 | 15 | 65252374 | 0.32 | 4088 | intron |  |  |
|  | rs2118610 | 15 | 65215388 | 0.31 | 4088 | intron |  |  |
|  | rs2118611 | 15 | 65188520 | 0.23 | 4088 | intron |  |  |
|  | rs2414937 | 15 | 65219730 | 0.18 | 4088 | intron |  |  |
|  | rs3743343 | 15 | 65273829 | 0.23 | 4088 | 3UTR |  |  |
|  | rs3825977 | 15 | 65268302 | 0.26 | 4088 | intron |  |  |
|  | rs4147358 | 15 | 65215068 | 0.33 | 4088 | intron |  |  |
|  | rs4601989 | 15 | 65239008 | 0.30 | 4088 | intron |  |  |
|  | rs4776890 | 15 | 65180099 | 0.30 | 4088 | intron |  |  |
|  | rs7163381 | 15 | 65201109 | 0.30 | 4088 | intron |  |  |
|  | rs7173811 | 15 | 65266105 | 0.43 | 4088 | intron |  |  |
|  | rs7174445 | 15 | 65238269 | 0.30 | 4088 | intron |  |  |
|  | rs7181556 | 15 | 65263822 | 0.33 | 4088 | intron |  |  |
|  | rs7183244 | 15 | 65248365 | 0.32 | 4088 | intron |  |  |
|  | rs731874 | 15 | 65233885 | 0.20 | 4088 | intron |  |  |
|  | rs745103 | 15 | 65222129 | 0.49 | 4088 | intron |  |  |
|  | rs750766 | 15 | 65225640 | 0.49 | 4088 | intron |  |  |
|  | rs9302242 | 15 | 65176466 | 0.45 | 4088 | intron |  |  |
|  | rs991157 | 15 | 65206067 | 0.20 | 4088 | intron |  |  |
|  | rs9972423 | 15 | 65178859 | 0.43 | 4088 | intron |  |  |
| Transcription factor 7-like 2 | rs1028629 | 10 | 114902646 | 0.20 | 6934 | intron |  |  |
|  | rs1056877 | 10 | 114915748 | 0.01 | 6934 | 3UTR |  |  |
|  | rs10749128 | 10 | 114855479 | 0.23 | 6934 | intron |  |  |
|  | rs10885399 | 10 | 114716167 | 0.28 | 6934 | intron |  |  |
|  | rs10885409 | 10 | 114798062 | 0.49 | 6934 | intron |  |  |
|  | rs10885410 | 10 | 114814463 | 0.20 | 6934 | intron |  |  |
|  | rs11196175 | 10 | 114726604 | 0.35 | 6934 | intron |  |  |
|  | rs11196203 | 10 | 114795850 | 0.14 | 6934 | intron |  |  |
|  | rs11196218 | 10 | 114830484 | 0.38 | 6934 | intron |  |  |
|  | rs11196224 | 10 | 114845387 | 0.38 | 6934 | intron |  |  |
|  | rs11196229 | 10 | 114856162 | 0.13 | 6934 | intron |  |  |
|  | rs12184389 | 10 | 114829760 | 0.17 | 6934 | intron |  |  |
|  | rs1225404 | 10 | 114904655 | 0.16 | 6934 | intron |  |  |
|  | rs12255372 | 10 | 114798892 | 0.34 | 6934 | intron |  |  |
|  | rs12573128 | 10 | 114720787 | 0.21 | 6934 | intron |  |  |
|  | rs12772424 | 10 | 114870541 | 0.45 | 6934 | intron |  |  |
|  | rs1362943 | 10 | 114878598 | 0.23 | 6934 | intron |  |  |
|  | rs1555485 | 10 | 114902524 | 0.08 | 6934 | intron |  |  |
|  | rs176632 | 10 | 114901069 | 0.07 | 6934 | intron |  |  |
|  | rs17685538 | 10 | 114787461 | 0.05 | 6934 | intron |  |  |
|  | rs2094405 | 10 | 114705679 | 0.38 | 6934 | intron |  |  |
|  | rs290481 | 10 | 114913815 | 0.21 | 6934 | intron |  |  |
|  | rs290483 | 10 | 114905204 | 0.22 | 6934 | intron |  |  |
|  | rs290487 | 10 | 114899721 | 0.20 | 6934 | intron |  |  |
|  | rs290489 | 10 | 114897045 | 0.36 | 6934 | intron |  |  |
|  | rs290491 | 10 | 114894524 | 0.16 | 6934 | intron |  |  |
|  | rs290494 | 10 | 114875861 | 0.11 | 6934 | intron |  |  |
|  | rs3750805 | 10 | 114837133 | 0.16 | 6934 | intron |  |  |
|  | rs3814570 | 10 | 114698500 | 0.24 | 6934 | flanking_5UTR |  |  |
|  | rs3814572 | 10 | 114837713 | 0.15 | 6934 | intron |  |  |
|  | rs3814573 | 10 | 114888083 | 0.38 | 6934 | intron |  |  |
|  | rs4917646 | 10 | 114892801 | 0.29 | 6934 | intron |  |  |
|  | rs4918789 | 10 | 114811797 | 0.50 | 6934 | intron |  |  |
|  | rs4918796 | 10 | 114870332 | 0.29 | 6934 | intron |  |  |
|  | rs7079711 | 10 | 114735778 | 0.13 | 6934 | intron |  |  |
|  | rs7080044 | 10 | 114889105 | 0.37 | 6934 | intron |  |  |
|  | rs7081062 | 10 | 114730735 | 0.33 | 6934 | intron |  |  |
|  | rs7085532 | 10 | 114849453 | 0.28 | 6934 | intron |  |  |
|  | rs7094463 | 10 | 114701973 | 0.41 | 6934 | intron |  |  |
|  | rs749596 | 10 | 114837895 | 0.10 | 6934 | intron |  |  |
|  | rs7901695 | 10 | 114744078 | 0.36 | 6934 | intron |  |  |
|  | rs7903424 | 10 | 114894026 | 0.27 | 6934 | intron |  |  |
|  | rs7908486 | 10 | 114824488 | 0.35 | 6934 | intron |  |  |
|  | rs7917983 | 10 | 114722872 | 0.42 | 6934 | intron |  |  |
| Thrombomodulin | rs1042580 | 20 | 22975621 | 0.47 | 7056 | 3UTR |  |  |
|  | rs1962 | 20 | 22974496 | 0.20 | 7056 | 3UTR |  |  |
| TIMP metallopeptidase inhibitor 2 | rs11077399 | 17 | 74375770 | 0.30 | 7077 | intron |  |  |
|  | rs12452379 | 17 | 74427053 | 0.44 | 7077 | intron |  |  |
|  | rs12600817 | 17 | 74413060 | 0.46 | 7077 | intron |  |  |
|  | rs2003241 | 17 | 74396712 | 0.09 | 7077 | intron |  |  |
|  | rs2009196 | 17 | 74382176 | 0.20 | 7077 | intron |  |  |
|  | rs3744787 | 17 | 74405266 | 0.15 | 7077 | intron |  |  |
|  | rs4789860 | 17 | 74410228 | 0.44 | 7077 | intron |  |  |
|  | rs7218237 | 17 | 74383233 | 0.11 | 7077 | intron |  |  |
|  | rs7342880 | 17 | 74386107 | 0.05 | 7077 | intron |  |  |
|  | rs7502935 | 17 | 74390224 | 0.30 | 7077 | intron |  |  |
|  | rs8068674 | 17 | 74419040 | 0.39 | 7077 | intron |  |  |
|  | rs9905930 | 17 | 74371511 | 0.16 | 7077 | intron |  |  |
|  | rs9909232 | 17 | 74366964 | 0.20 | 7077 | intron |  |  |
| Transmembrane and coiled-coil domains 6 | rs12517200 | 5 | 140000105 | 0.31 | 55374 | intron |  |  |
|  | rs3822356 | 5 | 140002620 | 0.19 | 55374 | intron |  |  |
|  | rs753279 | 5 | 140004002 | 0.44 | 55374 | intron |  |  |
|  | rs753280 | 5 | 140004226 | 0.41 | 55374 | intron |  |  |
| Tumor necrosis factor | rs1799964 | 6 | 31650287 | 0.20 | 4049 | flanking_3UTR |  |  |
|  | rs1800630 | 6 | 31650455 | 0.15 | 4049 | flanking_3UTR |  |  |
|  | rs3093664 | 6 | 31652621 | 0.09 | 7124 | intron |  |  |
| Tumor necrosis factor receptor superfamily, member 1A | rs1800693 | 12 | 6310270 | 0.43 | 7132 | intron |  |  |
|  | rs4149577 | 12 | 6317783 | 0.47 | 7132 | intron |  |  |
|  | rs4149578 | 12 | 6317698 | 0.08 | 7132 | intron |  |  |
| Tumor protein p53 | rs12951053 | 17 | 7518132 | 0.09 | 7157 | intron |  |  |
|  | rs2078486 | 17 | 7583083 | 0.19 | 7157 | intron |  |  |
| Tripartite motif containing 54 | rs4665962 | 2 | 27368609 | 0.25 | 57159 | intron |  |  |
|  | rs4665963 | 2 | 27382196 | 0.31 | 57159 | intron |  |  |
| Resistance to inhibitors of cholinesterase 3 homolog | rs1055233 | 11 | 8084071 | 0.31 | 7275 | 3UTR |  |  |
|  | rs10769866 | 11 | 8032727 | 0.31 | 7275 | intron |  |  |
|  | rs11041723 | 11 | 8035280 | 0.38 | 7275 | intron |  |  |
|  | rs11041740 | 11 | 8074094 | 0.18 | 7275 | intron |  |  |
|  | rs11601971 | 11 | 8042228 | 0.11 | 7275 | intron |  |  |
|  | rs11823329 | 11 | 8037442 | 0.15 | 7275 | intron |  |  |
|  | rs1406095 | 11 | 8051258 | 0.39 | 7275 | intron |  |  |
|  | rs16935778 | 11 | 8077177 | 0.06 | 7275 | intron |  |  |
|  | rs1881233 | 11 | 8019745 | 0.16 | 7275 | intron |  |  |
|  | rs1881240 | 11 | 8048000 | 0.36 | 7275 | intron |  |  |
|  | rs2242501 | 11 | 8074679 | 0.23 | 7275 | intron |  |  |
|  | rs2242503 | 11 | 8075048 | 0.28 | 7275 | intron |  |  |
|  | rs2280726 | 11 | 8039305 | 0.23 | 7275 | intron |  |  |
|  | rs3750955 | 11 | 8082818 | 0.20 | 7275 | 3UTR |  |  |
|  | rs3752898 | 11 | 8073496 | 0.17 | 7275 | intron |  |  |
|  | rs3794018 | 11 | 8054010 | 0.43 | 7275 | intron |  |  |
|  | rs4385931 | 11 | 8066224 | 0.28 | 7275 | intron |  |  |
|  | rs4575312 | 11 | 8065299 | 0.26 | 7275 | intron |  |  |
|  | rs4758040 | 11 | 8057749 | 0.37 | 7275 | intron |  |  |
|  | rs4758287 | 11 | 8056489 | 0.25 | 7275 | intron |  |  |
|  | rs6578921 | 11 | 8051078 | 0.49 | 7275 | intron |  |  |
|  | rs7480804 | 11 | 8066385 | 0.04 | 7275 | intron |  |  |
|  | rs7926428 | 11 | 8055693 | 0.13 | 7275 | intron |  |  |
|  | rs7926527 | 11 | 8055765 | 0.35 | 7275 | intron |  |  |
|  | rs7932687 | 11 | 8045333 | 0.37 | 7275 | intron |  |  |
|  | rs7939737 | 11 | 8070170 | 0.04 | 7275 | intron |  |  |
| Tubby-like protein 1 | rs11754612 | 6 | 35584540 | 0.11 | 7287 | intron |  |  |
|  | rs12215920 | 6 | 35582051 | 0.21 | 7287 | intron |  |  |
|  | rs2064317 | 6 | 35585010 | 0.22 | 7287 | coding |  |  |
|  | rs2067997 | 6 | 35579224 | 0.09 | 7287 | intron |  |  |
|  | rs2273000 | 6 | 35586446 | 0.32 | 7287 | intron |  |  |
|  | rs7752728 | 6 | 35585130 | 0.24 | 7287 | intron |  |  |
|  | rs7769417 | 6 | 35581008 | 0.44 | 7287 | intron |  |  |
|  | rs9296155 | 6 | 35585281 | 0.20 | 7287 | intron |  |  |
| Urocortin 2 | rs13319651 | 3 | 48574845 | 0.06 | 90226 | 3UTR |  |  |
| Urocortin 3 | rs10795268 | 10 | 5398461 | 0.34 | 114131 | intron |  |  |
| Uncoupling protein 1 | rs12502572 | 4 | 141704584 | 0.39 | 7350 | intron |  |  |
|  | rs6536991 | 4 | 141701031 | 0.29 | 7350 | intron |  |  |
|  | rs6818140 | 4 | 141707405 | 0.18 | 7350 | intron |  |  |
|  | rs6822807 | 4 | 141707198 | 0.28 | 7350 | intron |  |  |
|  | rs7688743 | 4 | 141708245 | 0.19 | 7350 | intron |  |  |
| Uncoupling protein 2 | rs655717 | 11 | 73361863 | 0.44 | 7351 | flanking_3UTR |  |  |
| Uncoupling protein 3 | rs1685354 | 11 | 73391239 | 0.28 | 7352 | intron |  |  |
|  | rs2075577 | 11 | 73393190 | 0.48 | 7352 | coding |  |  |
|  | rs3741135 | 11 | 73391608 | 0.15 | 7352 | intron |  |  |
| Upstream transcription factor 1 | rs2073656 | 1 | 159277979 | 0.29 | 7391 | intron |  |  |
|  | rs2516837 | 1 | 159281351 | 0.43 | 7391 | intron |  |  |
|  | rs2774276 | 1 | 159278340 | 0.21 | 7391 | intron |  |  |
| Upstream transcription factor 2 | rs10405246 | 19 | 40460789 | 0.15 | 7392 | intron |  |  |
| Urotensin 2 | rs170629 | 1 | 7832978 | 0.25 | 10911 | intron |  |  |
|  | rs228651 | 1 | 7833686 | 0.41 | 10911 | intron |  |  |
| Vitamin D receptor | rs10783219 | 12 | 46581755 | 0.34 | 7421 | intron |  |  |
|  | rs10875695 | 12 | 46579304 | 0.24 | 7421 | intron |  |  |
|  | rs11168267 | 12 | 46537809 | 0.12 | 7421 | intron |  |  |
|  | rs11168287 | 12 | 46571681 | 0.48 | 7421 | intron |  |  |
|  | rs11168293 | 12 | 46579983 | 0.31 | 7421 | UTR |  |  |
|  | rs1544410 | 12 | 46526102 | 0.42 | 7421 | intron |  |  |
|  | rs2107301 | 12 | 46541837 | 0.26 | 7421 | intron |  |  |
|  | rs2189480 | 12 | 46550095 | 0.34 | 7421 | intron |  |  |
|  | rs2238136 | 12 | 46563980 | 0.26 | 7421 | intron |  |  |
|  | rs2239179 | 12 | 46544033 | 0.42 | 7421 | intron |  |  |
|  | rs2239180 | 12 | 46542313 | 0.14 | 7421 | intron |  |  |
|  | rs2239182 | 12 | 46541678 | 0.48 | 7421 | intron |  |  |
|  | rs2239186 | 12 | 46555677 | 0.14 | 7421 | intron |  |  |
|  | rs2254210 | 12 | 46559981 | 0.30 | 7421 | intron |  |  |
|  | rs2853564 | 12 | 46564754 | 0.36 | 7421 | intron |  |  |
|  | rs3782905 | 12 | 46552434 | 0.32 | 7421 | intron |  |  |
|  | rs4760648 | 12 | 46566932 | 0.43 | 7421 | intron |  |  |
|  | rs7299460 | 12 | 46582535 | 0.35 | 7421 | intron |  |  |
|  | rs7305032 | 12 | 46536127 | 0.35 | 7421 | intron |  |  |
|  | rs757343 | 12 | 46525942 | 0.15 | 7421 | intron |  |  |
|  | rs886441 | 12 | 46549231 | 0.25 | 7421 | intron |  |  |
| UTR: untranslated region |  |  |  |  |  |  |  |  |
